# Building Blocks and COFs Formed *in Concert*—Three‐Component Synthesis of Pyrene‐Fused Azaacene Covalent Organic Framework in the Bulk and as Films

**DOI:** 10.1002/anie.202302872

**Published:** 2023-06-13

**Authors:** Laura Frey, Orlando Oliveira, Ashish Sharma, Roman Guntermann, Soraia P. S. Fernandes, Krystal M. Cid‐Seara, Hosanna Abbay, Henry Thornes, João Rocha, Markus Döblinger, Tim Kowalczyk, Akshay Rao, Laura M. Salonen, Dana D. Medina

**Affiliations:** ^1^ Department of Chemistry and Center for Nanoscience (CeNS) Ludwig-Maximilians-University Butenandtstraße 11 (E) 81377 Munich Germany; ^2^ CINBIO Universidade de Vigo Department of Organic Chemistry 36310 Vigo Spain; ^3^ International Iberian Nanotechnology (INL) Avenida Mestre José Veiga 4715-330 Braga Portugal; ^4^ CICECO—Aveiro Institute of Materials University of Aveiro 3810-193 Aveiro Portugal; ^5^ Cavendish Laboratory University of Cambridge 19 JJ Thomson Avenue Cambridge CB3 0HE UK; ^6^ Associate Laboratory for Green Chemistry-Network of Chemistry and Technology (LAQV-REQUIMTE) University of Aveiro Campus Universitário de Santiago 3810-193 Aveiro Portugal; ^7^ Department of Inorganic Chemistry University of Vigo Campus Universitário, As Lagoas-Marcosende 36310 Vigo Spain; ^8^ Department of Chemistry and Advanced Materials Science and Engineering Center (AMSEC) Western Washington University 516 High Street Bellingham WA-98225 USA

**Keywords:** Covalent Organic Frameworks, Layer Alignment, Optical Properties, Pyrene-Fused Azaacenes, Three-Component Synthesis

## Abstract

A three‐component synthesis methodology is described for the formation of covalent organic frameworks (COFs) containing extended aromatics. Notably, this approach enables synthesis of the building blocks and COF along parallel reaction landscapes, on a similar timeframe. The use of fragmental building block components, namely pyrene dione diboronic acid as aggregation‐inducing COF precursor and the diamines *o*‐phenylenediamine (Ph), 2,3‐diaminonaphthalene (Naph), or (1*R*,2*R*)‐(+)‐1,2‐diphenylethylenediamine (2Ph) as extending functionalization units in conjunction with 2,3,6,7,10,11‐hexahydroxytriphenylene, resulted in the formation of the corresponding pyrene‐fused azaacene, i.e., Aza‐COF series with full conversion of the dione moiety, long‐range order, and high surface area. In addition, the novel three‐component synthesis was successfully applied to produce highly crystalline, oriented thin films of the Aza‐COFs with nanostructured surfaces on various substrates. The Aza‐COFs exhibit light absorption maxima in the blue spectral region, and each Aza‐COF presents a distinct photoluminescence profile. Transient absorption measurements of Aza‐Ph‐ and Aza‐Naph‐COFs suggest ultrafast relaxation dynamics of excited‐states within these COFs.

## Graphical abstract

Significant limitations exist in the synthesis of highly ordered structures including the solubility, shape, and packing of building blocks. A three‐component synthesis allows for the preparation of novel pyrene‐fused azaacene covalent organic frameworks (COFs) in a one‐step reaction. The COFs can also be prepared as films, and the optical properties of the materials are studied.

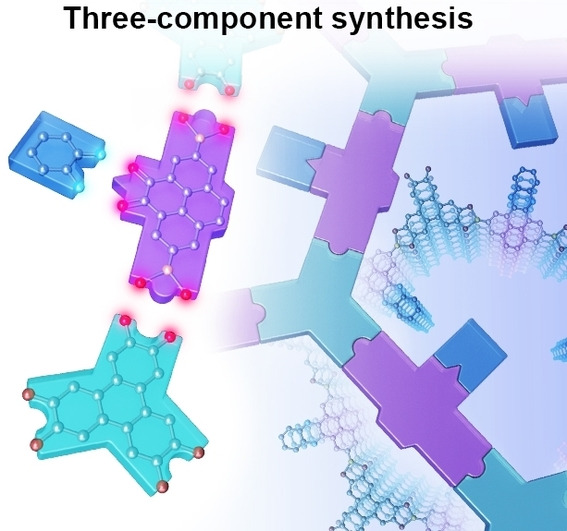

## Introduction

Molecular framework materials have attracted high interest owing to their unique construction principle, which allows for the precise positioning of molecular segments in a porous crystalline matrix. To tailor covalent organic frameworks (COFs) for a specific application, their chemical and physical properties are typically altered by using the extensive range of tools available in organic chemistry. There are two primary synthesis strategies: (*i)* pre‐functionalization of building blocks, where the desired functional groups are installed at specific positions of the building blocks prior to the COF assembly, and (*ii*) post‐synthetic modification (PSM), where assembled frameworks bearing anchoring groups react with specific chemical components to form a functional product with desired characteristics.^[1]^ To select the most suitable strategy for creating the desired framework, several factors need to be considered. For building block modification, solubility, geometry, and packing of the resulting building blocks are vital aspects that synergistically influence the formation of high‐quality COFs. For the PSM approach, the framework stability under the employed functionalization conditions and the reaction yield are among the primary considerations. In recent years, the three‐component synthesis has emerged as a powerful methodology for the synthesis of functional COFs.[^2^, ^3^] Early reports mainly focused on the synthesis of dual‐linkage COFs, which incorporate both imine and boronic ester linkages in their final structure.^[4]^ Subsequent studies explored the stabilization of reactive imine bonds. A prominent example is the synthesis of α‐aminonitrile‐ and quinoline‐linked COFs using one‐pot Strecker and Povarov syntheses.^[5]^

To integrate COF materials into devices, the synthesis of functional COFs as thin films is essential to enable their utilization as active deposited layers for specific applications, such as sensing,^[6]^ separation,^[7]^ and optoelectronics.^[8]^ For the latter, the assembly of layered 2D COFs onto conductive electrodes has been extensively investigated as a means to control the charge carrier percolation paths in the molecular segments related to the conducting surface.^[8]^ Several methods for thin‐film assembly with oriented 2D COF crystallites have been introduced, including solvothermal in situ growth,^[9]^ interfacial synthesis,^[10]^ and continuous‐flow synthesis.^[11]^ Because these synthesis approaches are based on growth through reactive precursor solutions, they are subject to similar limitations as bulk synthesis.

Linear acenes consisting of laterally fused benzenoid rings have been extensively studied owing to their intriguing properties related to energy conversion and optoelectronic applications.^[12]^ The most prominent example of the latter is the pentacene dimers, which feature an efficient singlet fission process.^[13]^ Such a process has been shown to take place also in acene‐containing COFs by theoretical means.^[14]^ However, acenes are labile materials with low solubility and their performance is highly dependent on their assembly. Therefore, bulky side groups are typically installed onto the acene backbone, which impart pentacene with greater stability and solubility while guiding their assembly.^[15]^ Additionally, substituted nitrogen‐doped acenes, known as azaacenes, are a class of robust acene analogs with fascinating photophysical properties, particularly as light‐emitting materials.[^13^, ^16^] Therefore, developing techniques to assemble these aromatic molecules in an ordered manner, as powders and films, is crucial for their characterization and practical utilization.[^2^, ^17^]

Very recently, we presented a PSM approach to incorporate pyrene‐fused azaacene moieties into a COF, using 2D Dione‐COF as precursor.^[18]^ This method achieved up to 33 % conversion of the dione moiety. To boost the yield of the conversion reaction and to expand the paradigm of COF synthesis, we introduce a three‐component synthesis approach, where the framework and functionalities are formed on a similar time scale. The presented strategy effectively addresses the challenges of low building block solubility and COF stability under post‐functionalization conditions.^[5]^ Notably, our approach also allows for the three‐component synthesis of functional frameworks as thin films, which had not been demonstrated with existing three‐component methodologies thus far.^[2]^

In practice, the pyrene dione building block^[19]^ was allowed to react with various diamines in the course of the COF formation to form the respective azaacenes. Impressively, the conversion of the dione moiety is quantitative, and the resulting COF powders, termed Aza‐Ph‐, Aza‐Naph‐, and Aza‐2Ph‐COF, feature accessible surface areas and long‐range order. All three COFs showed light absorption in the visible range extending to the near‐infrared (NIR) region. Aza‐Ph‐ and Aza‐Naph‐COF displayed two photoluminescence (PL) emission bands, whereas Aza‐2Ph‐COF exhibited one emission band. Moreover, highly oriented thin films of all three COFs were successfully grown on various substrates, with thicknesses ranging from 200 to 400 nm. Furthermore, transient absorption experiments indicated temporal dynamics of excited‐states within the investigated COFs.

## Results and Discussion

**Scheme 1:**
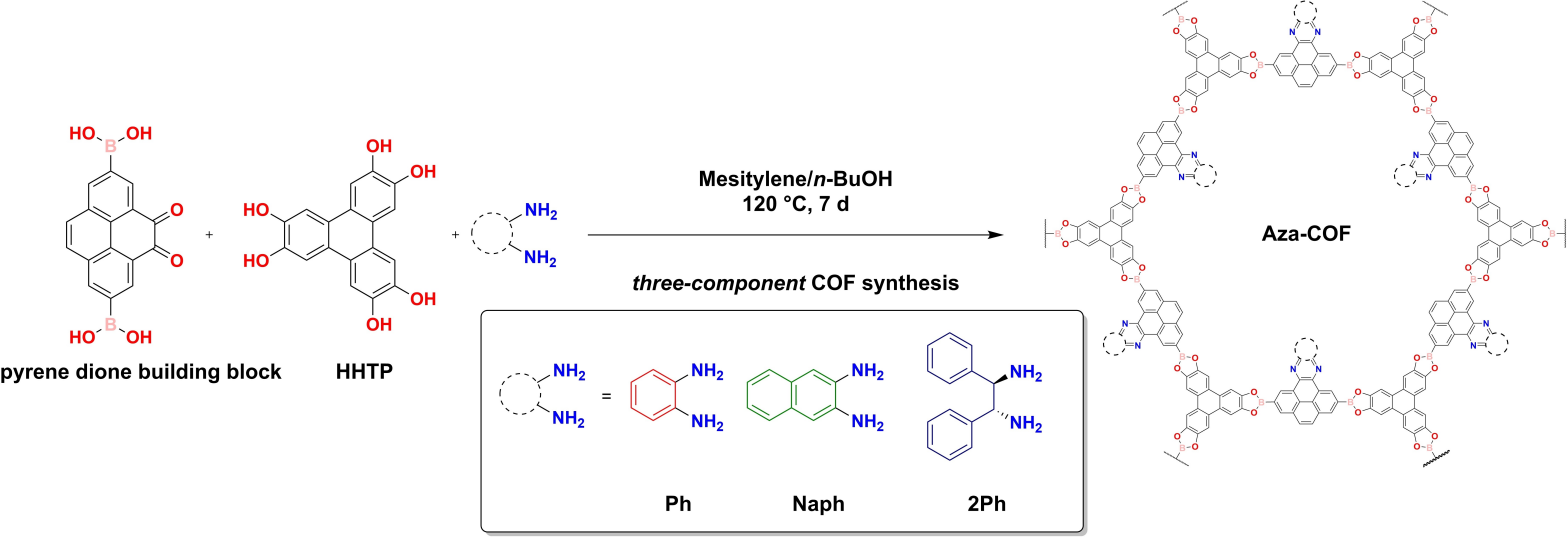
Schematic illustration of the three‐component synthesis of the Aza‐COF series using *o*‐phenylenediamine (Ph, red), 2,3‐diaminonaphthalene, (Naph, green), or (1*R*,2*R*)‐(+)‐1,2‐diphenylethylenediamine (2Ph, blue) as amines.

Pyrene dione building block and Dione‐COF were synthesized following our previously reported procedure.[^18^, ^19^] The three‐component COF synthesis was carried out under solvothermal conditions (Scheme 1). Briefly, in a 6 mL tube under argon, pyrene dione building block (1 equiv.) and 2,3,6,7,10,11‐hexahydroxytriphenylene (HHTP) (0.67 equiv.) were suspended in mesitylene and anhydrous *n*‐butanol 1 : 1 *v*/*v* along with 2.1 equiv. of the respective amine (either Ph, Naph, or 2Ph). The tube was sealed and placed in a pre‐heated oven for 7 days at 120 °C. After the given reaction time, a dark brown precipitate was obtained in all three cases. The powders were isolated by filtration and washed with anhydrous acetone to give Aza‐Ph‐, Aza‐Naph‐, and Aza‐2Ph‐COF powders (for further information, see the Supporting Information Section 2).

**Figure 1:**
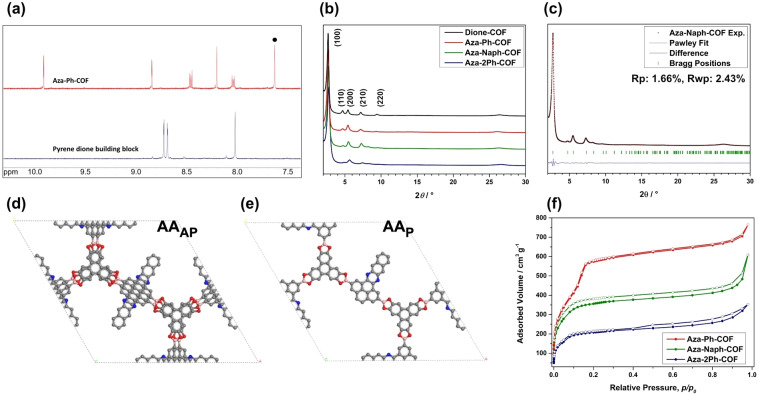
a) Overlay of ^1^H NMR spectrum of hydrolyzed Aza‐Ph‐COF (top) and pyrene dione building block (bottom) (400 MHz, (CD_3_)_2_SO+D_2_O). The peak marked with a black dot corresponds to the C−H proton of HHTP. b) PXRD patterns of Dione‐COF, Aza‐Ph‐, Aza‐Naph‐, and Aza‐2Ph‐COF. c) Experimental PXRD pattern (black dots), Pawley refinement (red line) and difference plot between the experimental data and the Pawley refined PXRD pattern (blue line) of Aza‐Naph‐COF. Bragg positions are indicated by green ticks. d), e) View of one unit cell for antiparallel AA_AP_ and parallel AA_P_ layer arrangement, respectively, using high symmetries for Aza‐Naph‐COF. f) Nitrogen sorption isotherms of Aza‐Ph‐, Aza‐Naph‐ and Aza‐2Ph‐COF, respectively.

To study the extent of the conversion of the dione moiety to the respective azaacene, the obtained powders were treated with DMSO‐*d*_6_ followed by an addition of D_2_O to completely hydrolyze the frameworks to the respective building blocks, and the obtained solutions were analyzed by ^1^H NMR spectroscopy (Figure S7–S9). After hydrolysis in all three cases, the absence of the doublets at 8.73 and 8.69 ppm and the singlet at 8.02 ppm of the pyrene dione building block unambiguously confirmed its complete reaction with the respective amines. In the case of Aza‐Ph‐COF (Figure 1a) and Aza‐Naph‐COF, the dione moieties were fully converted to the target azaacenes (Figures S7 and S8, respectively, Figure S11). In the case of Aza‐2Ph‐COF, a full reaction of the dione moieties resulted in a mixture of products, evident in the ^1^H NMR spectrum (Figure S9). As condensation of 2Ph with the dione moiety will first lead to a non‐aromatic imine, with subsequent dehydrogenation then yielding the desired azaacene product, we assumed the mixture to stem mainly from these species. To test this assumption, we bubbled air through the NMR solution, and indeed after 2 h mainly one product is obtained, which we attribute to the azaacene species (Figure S10). Furthermore, mass spectrometry confirmed the formation of this product.

The success of the condensation reaction between the pyrene dione building block and the diamines was also indicated by Fourier‐transform infrared (FTIR) spectroscopy. As shown in Figure S17, an absorption band at ≈1668 cm^−1^ corresponding to the carbonyl group stretching was observed for Dione‐COF. In contrast, this band was not detected in the spectra of Aza‐Ph‐, Aza‐Naph‐, and Aza‐2Ph‐COF, indicating the successful reaction of the dione moieties with the diamines. In addition, we observed new or intensified bands at 1404 (Aza‐Ph‐ and Aza‐Naph‐COF) and 1412 cm^−1^ (Aza‐2Ph‐COF), at 1238 cm^−1^, and 1099 cm^−1^ (all Aza‐COFs), which were attributed to stem from C=C and C=N stretching, skeletal, and C−H vibrations of the azaacene ring.^[20]^

Subsequently, the obtained powders were analyzed by powder X‐ray diffraction (PXRD) (Figure 1b). In all cases, the three‐component reaction yielded highly ordered crystalline materials that can be fully indexed in agreement with the Dione‐COF lattice parameters.^[19]^ A close look at the diffraction patterns reveals that the relative intensity corresponding to the (110) and (200) reflections (I_110_ and I_200_) changes upon lateral extension of the aromatic system of the pyrene dione. For the non‐modified Dione‐COF, I_110_ and I_200_ are similar. In contrast, for Aza‐Ph, Aza‐Naph‐, and Aza‐2Ph‐COF, I_110_ is greatly diminished compared to I_200_. This is attributed to the introduction of scattering elements into the pore along the 200 plane (Figure S18). Such pore filling can be related to successful conversion of the dione moieties.^[21]^

Furthermore, for the different pyrene substitutions, the reflection associated with the interlayer distance showed variation. For Dione‐COF, the corresponding reflection was observed at 26.3° 2*θ*, corresponding to a π‐stacking distance of about 3.4 Å. For the modified Aza‐COFs, shifted reflections at 26.1°, 26.2°, and 26.6° 2*θ* were obtained for Aza‐Ph, Aza‐Naph‐, and Aza‐2Ph‐COF, respectively, corresponding to π‐stacking distances of 3.4 Å in the case of Aza‐Ph‐ and Aza‐Naph‐COF and 3.3 Å for Aza‐2Ph‐COF. Interestingly, a tighter interlayer distance was observed for the most sterically demanding Aza‐2Ph‐COF as compared to the Aza‐COFs extended in a planar manner.

To relate these observations to the COF layer packing and to ultimately shed light on the overall COF structure, we turned to COF structure modeling by geometry optimization and force field calculations^[22]^ combined with PXRD simulation. In the case of unilateral building blocks, such as pyrene dione or the pyrene‐fused azaacenes thereof, we identify two general modes of building block stacking arrangements in successive layers: (i) parallel, where building blocks are fully overlapping, AA_P,_ and (ii) antiparallel, AA_AP_, where the moieties alternate in an antiparallel manner between successive layers (Figures S18). COF structure models were simulated corresponding to the possible AA_P_ and AA_AP_ packing scenarios (Figure 1d, e and Figures S19–S21). For fully overlapping AA_P_ and antiparallel AA_AP_ Aza‐Ph‐ and Aza‐Naph‐COFs, *P* $\bar{6}$_2_ *m* and *P*6_3_ *cm* space groups of the hexagonal crystal system were employed, respectively.^[19]^ To simulate an AA_P_ layer arrangement for Aza‐2Ph‐COF, *P*1 was found to be suitable to describe the phenyl groups rotating out of plane.

The calculated PXRD patterns obtained for the modeled COFs were compared to those obtained experimentally (Figure S19–S21 for Aza‐Ph‐COF, Aza‐Naph‐COF, and Aza‐2Ph‐COF, respectively). Because of very similar periodicities within the 2D layers, the calculated PXRD patterns of all models show essentially the same reflection positions at low angles as observed experimentally (Figure S19–S21). However, the different models result in different relative reflection intensities, especially for I_110_ and I_200_. For all COFs, the AA_AP_ model reproduced well the observed weakening of I_110_ upon pore occupancy.

Employing hexagonal symmetry space groups for AA models results in a systematic and alternating distribution of the azaacene moieties pointing to neighboring pores. In this arrangement, every pore incorporates three azaacene moieties. To simulate a different distribution, COF structures with AA_P_ layer assembly featuring three adjacent azaacene moieties in the pores were simulated in a *P*1 symmetry (Figure S19–S21). Here, the resulting calculated PXRD patterns also reproduce well the relative reflection intensity obtained experimentally, particularly in the case of Aza‐2Ph‐ and Aza‐Ph‐COFs. Other distribution schemes failed to reproduce the experimentally obtained diffraction pattern (Figure S22).

The models for AA_P_ and AA_AP_ azaacene orientations have slightly different interlayer distances. The AA_P_ models resulted in interlayer distances of 3.5, 3.5, and 3.7 Å for Aza‐Ph‐, Aza‐Naph‐ and Aza‐2Ph‐COF, respectively, while the AA_AP_ structure models yield shorter interlayer distances (about 3.4, 3.4, and 3.3 Å, for Aza‐Ph‐, Aza‐Naph‐ and Aza‐2Ph‐COF, respectively). Evidently, the latter are in good agreement with experimentally observed PXRD patterns.

The interlayer spacing depends among other factors on the stacking offset, owing to the degree of π‐electron cloud overlap and repulsion.^[23]^ Therefore, different slip‐stacked models for the AA_P_ structure models with three adjacent azaacene moieties in the pore were constructed. In addition, the effect of the orientations of the azaacenes across the pores and the lattice energy of the respective models were calculated by self‐consistent charge density‐functional tight‐binding (DFTB) (Table S1).^[24]^ In the simulations, COFs with AA_P_ slip‐stacked layer structures afforded the lowest lattice energy for Aza‐Ph‐COF and Aza‐Naph‐COF. The generated PXRD patterns agree well with the experimental ones both in low and high diffraction angle regimes for Aza‐Ph‐COF. In the case of Aza‐Naph‐COF, however, although the calculated lattice energy points to AA_P_ as the favored layer arrangement, the simulated PXRD pattern of the respective AA_P_ slip‐stacked model features different relative reflection intensities than those found in the experimental pattern. In contrast, for Aza‐2Ph‐COF, the lattice energies suggest a slight preference for AA_AP_ compared with other AA layer arrangements. These findings suggest that the Aza‐Ph‐COF is likely to be of AA_P_ character whereas Aza‐Naph‐ and Aza‐2Ph‐COFs are of AA_AP_ character. That said, deviations from these ideal stacking schemes where both AA_AP_ and AA_P_ are present, to some extent, in the crystalline matrix are to be expected, constituting a mixed AA_AP/P_ layer arrangement in practice (Figure S22). In addition, the DFTB models predict negligible impact on the lattice energy of the distribution of azaacenes jutting into the pores. Accordingly, we postulate that variation in the orientations of the azaacene moieties in the pore might be anticipated, giving rise to an average structure with scattering elements largely centered along the (200) plane.

To optimize the unit cell parameters further, we utilized the antiparallel structure model of high symmetry of the Aza‐2Ph‐ and Aza‐Naph‐COFs and the slip‐stacked AA_P_ model for the Aza‐Ph‐COF. The obtained unit cell paraments were refined using the Pawley method according to the observed PXRD patterns (Figure S23–24). Thereby, unit cell parameters of *a*=*b*=37.5 Å, *c*=6.9 Å, *α*=*β*=90° and *γ*=120° were obtained for Aza‐Ph‐COF, *a*=*b*=37.4 Å, *c*=6.8 Å, *α*=90°, *β*=89.97° and *γ*=120° for Aza‐Naph‐COF and *a*=*b*=37.3 Å, *c*=6.8 Å, *α*=*β*=90° and *γ*=120° for Aza‐2Ph‐COF, agreeing well with the unit cell parameters of Dione‐COF.^[19]^

We postulated that the speed of the cyclocondensation reaction between the pyrene dione moiety and the diamine could affect the mode of AA layer assembly in the Aza‐COFs. Previously, we proposed the pyrene dione moieties in Dione‐COF to assemble in an antiparallel manner to increase intermolecular interactions within the COF layers.^[19]^ To elaborate on this aspect, we first studied the reaction of the pyrene dione building block with Ph in the presence of catechol as a mimic of HHTP at room temperature in a mixture of deuterated mesitylene and deuterated *n*‐butanol (1 : 1 *v*/*v*) by NMR spectroscopy (Figure S12). Interestingly, the pyrene dione building block remains unreacted upon addition of catechol (2.2 equiv.) to the solution (Figure S12, middle). However, after subsequent addition of Ph (2.2 equiv.), the ^1^H NMR spectrum evidenced instant conversion of the dione to the corresponding azaacene in quantitative yield (Figure S12, bottom). The rapidity of the azaacene formation reaction at room temperature suggests that in the case of three‐component Aza‐Ph‐COF synthesis, the azaacene formation occurs more quickly than the COF formation, the latter typically spanning from several hours to days at elevated temperatures. Accordingly, we argue that the pyrene‐fused azaacene is the main component constructing the Aza‐Ph‐COF rather than the pyrene dione or a mixture of building blocks. This condition may direct the assembly towards AA_P_ by attenuating the impact of the strong dipole moment of pyrene dione on the layer assembly. Next, to assess the relative rapidity of the azaacene formation reactions, reactions between pyrene‐4,5‐dione and Ph or Naph at room temperature in a mixture of deuterated mesitylene and deuterated *n*‐butanol (1 : 1 *v*/*v*) were followed by ^1^H NMR spectroscopy. Interestingly, upon addition of Ph, conversion of pyrene‐4,5‐dione to the corresponding azaacene was observed in 10 % yield, increasing to 66 % after 1 h (Figure S13). The addition of catechol was not found to influence the yield of the reaction (Figure S15). In the case of Naph, the reaction proceeded much more slowly, with negligible conversion observed upon addition, and merely 13 % after 1 h (Figure S14). The slower reaction profile of Naph could stem from its lower solubility as compared to Ph under the employed conditions. Practically, such slower azaacene formation in the case of Naph may give rise to a multi‐component COF synthesis situation, in which pyrene dione and azaacene building blocks coexist for an extended period of time, subsequently leading to a complex layer assembly in the case of Aza‐Naph‐COF.

The impact of the azaacene modification on the pore accessibility of the COFs was analyzed using nitrogen sorption at 77 K (Figures 1f, S25). For Aza‐Ph‐COF, a type IV isotherm (Figure 1f, red curve) was obtained, characteristic of mesoporous materials, with two steep and well‐defined nitrogen uptakes at relatively low partial pressure (*p*/*p*_0_ *<*0.16 and up to 567 cm^3^ g^−1^).^[25]^ For Aza‐Naph‐ and Aza‐2Ph‐COF sharp nitrogen uptakes at low partial pressure are obtained (*p*/*p*_0_<0.12 and up to 366 cm^3^ g^−1^ for Aza‐Naph‐ and *p*/*p*_0_<0.11 and up to 195 cm^3^ g^−1^ for Aza‐2Ph‐COF, respectively). BET surface areas of 1834 m^2^ g^−1^, 1375 m^2^ g^−1^, and 866 m^2^ g^−1^ were obtained for Aza‐Ph‐, Aza‐Naph‐, and Aza‐2Ph‐COF, respectively. Pore size distributions were calculated using the QSDFT model for cylindrical pores (adsorption branch) (Figure S26), and pore sizes of 2.7 nm, 2.0 nm, and 2.2 nm were obtained for Aza‐Ph‐, Aza‐Naph‐ and Aza‐2Ph‐COF, respectively, which are in good agreement with the structure simulations.

**Figure 2:**
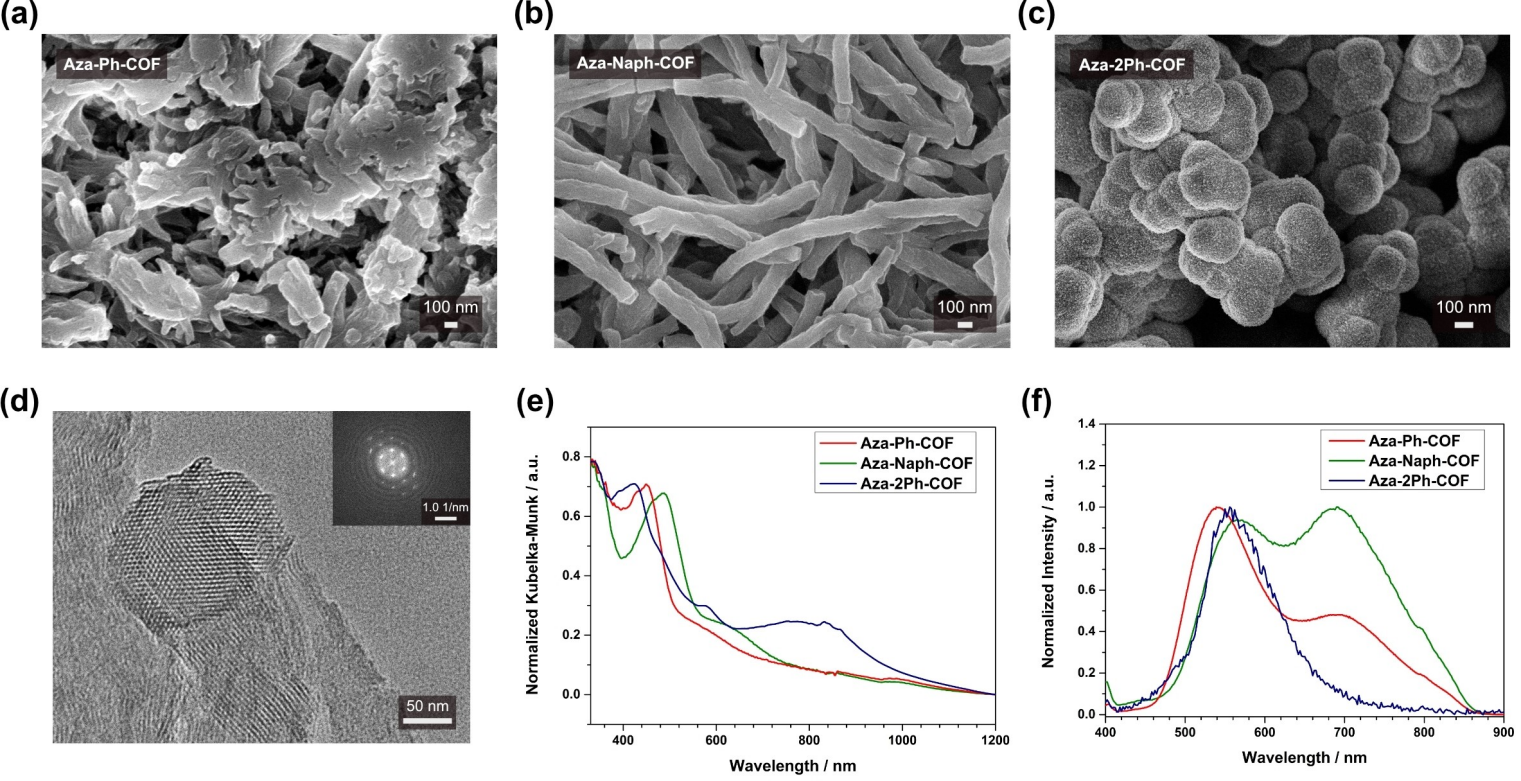
a), b), and c) SEM images of Aza‐Ph‐, Aza‐Naph‐, and Aza‐2Ph‐COF powders, respectively. d) TEM image of Aza‐Ph‐COF. The inset shows a power spectrum of the image. e) Normalized absorption spectra and f) normalized PL emission spectra of Aza‐Ph‐ (red), Aza‐Naph‐ (green), and Aza‐2Ph‐COF (blue) powders.

Scanning electron microscopy (SEM) analysis of the COF powders revealed that the wire‐like morphology of Dione‐COF^[19]^ is maintained upon modification of the framework in the case of Aza‐Ph‐ and Aza‐Naph‐COF (Figure 2a, b). Aza‐2Ph‐COF has a substantially different sphere‐like morphology consisting of small crystallites (Figure 2c). Transmission electron microscopy (TEM) analysis of the COFs further evidences their highly crystalline nature (Figure 2d, S27), and projections along the *c*‐axis reveal the expected honeycomb structure in all cases. Power spectra of the obtained images reveal higher order peaks, reflecting the high crystallinity and long‐range order of the crystallites (Figure 2d, S27). Thermogravimetric analysis (TGA) under synthetic air flow showed that all three Aza‐COFs are thermally stable up to 500 °C, at which point a significant weight loss was observed, corresponding to degradation of the COF materials (Figure S28).

**Figure 3:**
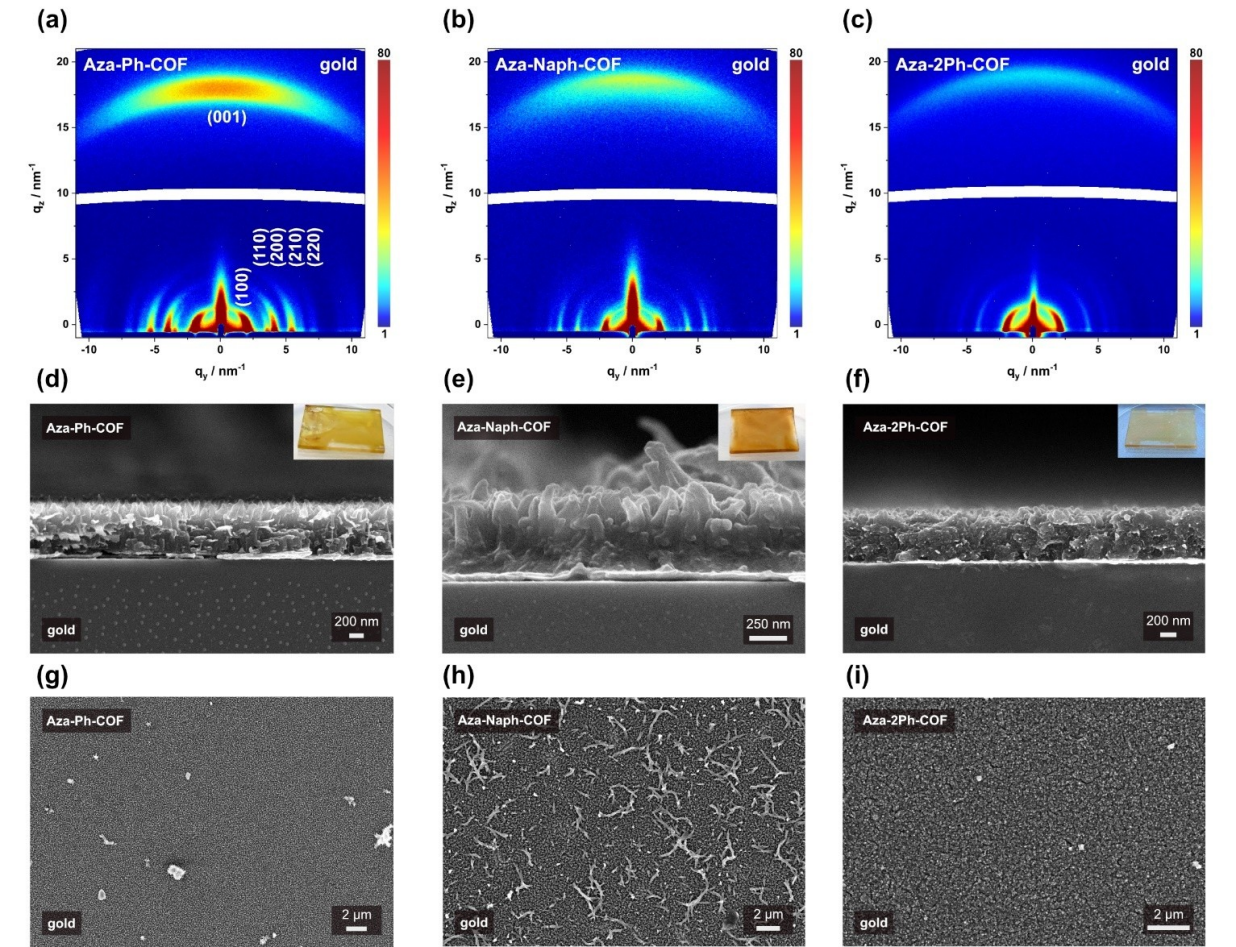
a), b), c) GIWAXS 2D patterns of Aza‐Ph‐, Aza‐Naph‐, and Aza‐2Ph‐COF thin films, respectively, grown on gold‐coated glass substrates. d), g), e, h), and f), i) SEM cross‐section and top‐view images of Aza‐Ph‐, Aza‐Naph‐, and Aza‐2Ph‐COF thin films, respectively, grown on gold‐coated glass substrates. The insets show macroscopic photographs of Aza‐Ph‐, Aza‐Naph‐, and Aza‐2Ph‐COF thin films on quartz substrates.

Processing of framework materials into coatings and films is challenging due to the low solubility of the obtained microcrystalline powders. Here, oriented COF thin films of Dione‐COF and the derived Aza‐COFs were synthesized on various substrates. The synthesis was carried out in glass vessels equipped with a Teflon substrate holder using the non‐epitaxy in situ approach and the solvothermal synthesis protocol for the Dione‐COF and the newly developed three‐component synthesis for the Aza‐COFs. The substrates were positioned horizontally using the Teflon substrate holder in the reaction vessel, immersed in the appropriate reactive precursor solution (including 2.1 equivalents of the respective amine for the synthesis of the Aza‐COF thin films). Subsequently, the reaction vessel was heated to 120 °C for 6 days. After removal from the reaction mixture, the substrate was thoroughly washed with anhydrous acetone and dried under a stream of nitrogen. Thereby, a homogeneous and shimmering layer (Figure 3d–f, insets) was obtained on top of different substrates, such as quartz or gold‐coated glass substrates. SEM cross‐section and top‐view images showed that a homogeneous coverage of the substrate was obtained with nanostructured surfaces consisting of individual, rod‐shaped crystallites preferentially oriented orthogonally to the surface for Dione‐, Aza‐Ph‐, and Aza‐Naph‐COF (Figure 3d–i, S29).

The crystallinity and crystal orientation were confirmed using grazing incidence wide‐angle X‐ray scattering (GIWAXS) (Figure 3a–c, S29). For all three Aza‐COF thin films and the Dione‐COF thin film, strong distinct reflections at *q*_y_=1.9, 3.4, 3.9, 5.1, 6.8 nm^−1^ and *q*_z_=18.6 nm^−1^ were obtained, corresponding to *hkl* (100), (110), (200), (210), (220), and (001). The *q* values obtained by data reduction to 1D plots match those obtained for the bulk powder XRD patterns. The GIWAXS data of all the studied COFs confirm the preferential orientation of the COF crystallites on the substrate with the [001] axis being oriented orthogonally to the substrate (Figure 3a–c). Importantly, the obtained 1D plots of the Aza‐COF films show (Figure S30) similar intensity ratios of the (110) and (200) reflections upon modification as the bulk, strongly indicative of successful azaacene formation.

The effect of the functionalization on the photophysical properties of the respective COF bulk powders and thin films was analyzed using UV/Vis and PL spectroscopies. The main absorption bands are located in the UV and blue spectral regions for Aza‐Ph‐, Aza‐Naph‐, and Aza‐2Ph‐COF bulk powders (Figure 2e, f). For Aza‐2Ph‐COF, a second broad absorption band is observed reaching the near‐infrared region. The main absorption maxima are offset with respect to each other, with Aza‐2Ph‐COF exhibiting the absorption maximum with the shortest wavelength at around 420 nm, followed by Aza‐Ph‐COF at approximately 450 nm, and Aza‐Naph‐COF with an absorption maximum at about 480 nm. This red‐shift in the absorption maxima can be related to the extension of the π‐conjugation in this series of COFs. A similar trend was observed for the absorbance measurements of the respective thin films on quartz substrates (Figure S31), indicating a successful conversion of the dione moieties in thin films using the newly developed three‐component approach. Steady‐state PL was measured for the three Aza‐COFs at an excitation wavelength of 378 nm. Aza‐Ph‐ and Aza‐Naph‐COF feature two emission bands that are red‐shifted respectively, in agreement with the absorption profiles. In contrast to the planar Aza‐Ph‐ and Aza‐Naph‐COF, in the case of Aza‐2Ph‐COF, one distinct emission band at around 555 nm was observed. Furthermore, a drastic change in the PL intensity is evident for the different COFs, where Aza‐Ph‐COF exhibits the highest intensity and Aza‐2Ph‐COF features the weakest PL emission. These findings are particularly interesting because the pristine Dione‐COF and the partially converted Dione‐COFs obtained by PSM are not PL active.^[18]^ However, in the case of fully converted Aza‐COFs, PL is observed.

**Figure 4:**
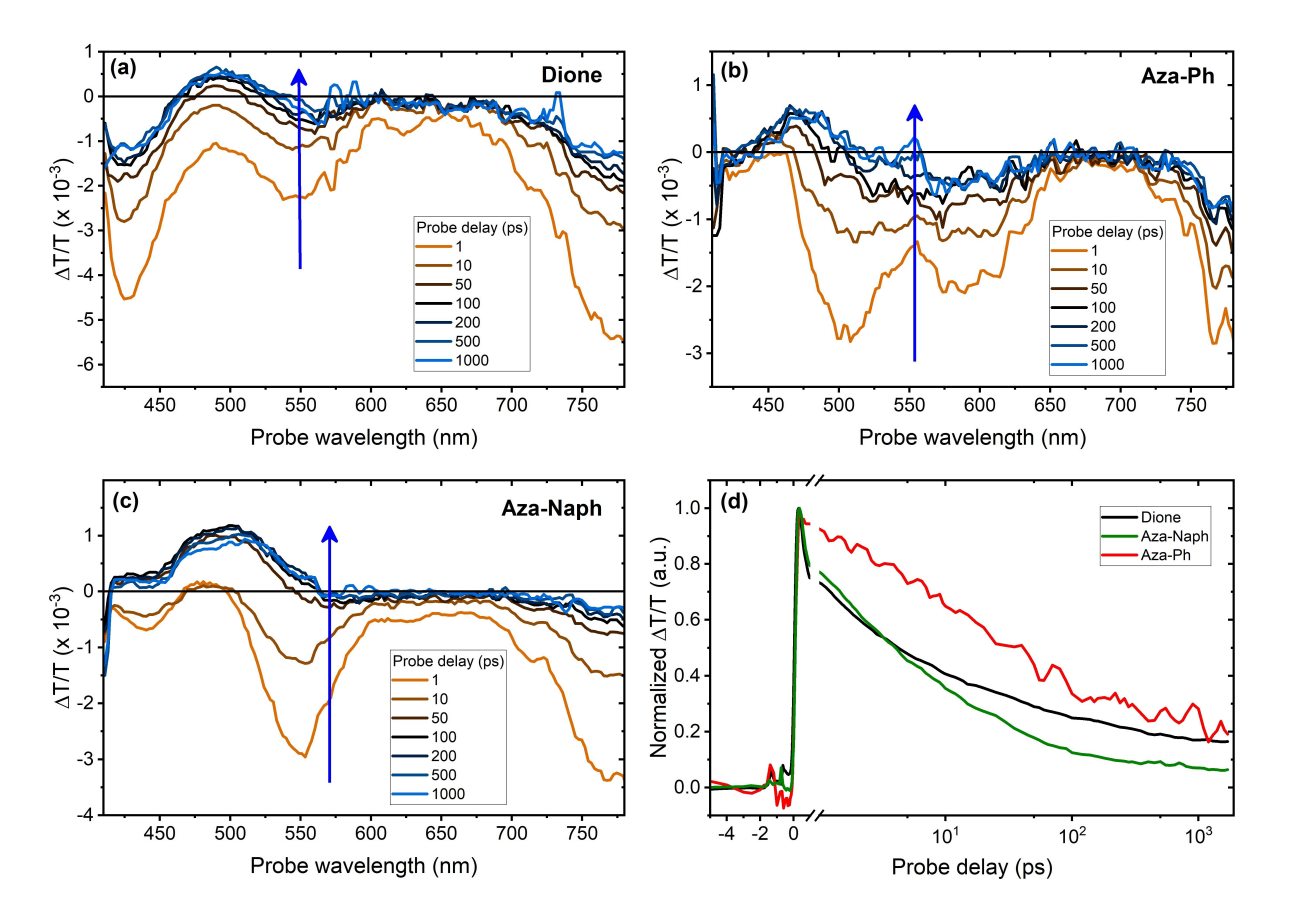
Sub‐ps transient absorption spectra of COFs. Transient absorption (TA) spectra of a) Dione‐, b) Aza‐Ph‐ and c) Aza‐Naph‐COFs for different delays of the probe. d) Comparison of the TA dynamics of the Dione‐COF (black line), Aza‐Naph‐ (green line), and Aza‐Ph‐COF (red line) monitored at 760–770 nm. Pump wavelength: 400 nm.

To unravel possible charge carrier dynamics particularly tracing non‐radiative relaxation channels in the Dione‐COF and linearly extended Aza‐COFs i.e., Aza‐Ph‐ and Aza‐Naph‐COF, we employed transient absorption (TA) at ultrafast (1 ps–1 ns) timescales. In TA, a pump pulse generates photoexcitations within the sample, which can then be probed at later times using a broadband probe pulse. A positive TA signal in Figure 4 correlates with an increase in transmission attributed to ground‐state bleaching (GSB), whereas a negative TA signal correlates with a decrease in transmission caused by photo‐induced absorption (PIA). Evidently, the TA spectra of all the three COFs involve broad PIA peaks spanning 400–750 nm for short (<10 ps) pump‐probe delays. For longer delays (>100 ps) of the probe, a recovery of the GSB feature can be observed for all the three COFs. We confirmed that the observed changes in the TA spectra are not due to exciton‐exciton annihilation or sample degradation (Figure S32–S33). Such changes in the TA spectra are reminiscent of exciton relaxation processes such as charge transfer (CT) in thin films of conjugated systems, where the initially generated exciton dissociates into free charge carriers or converts to a longer‐lived CT state.^[26]^ Comparing the TA dynamics of the three COFs (Figure 4d), it can be seen that exciton relaxation is significantly slower in Aza‐Ph‐COF in comparison to Aza‐Naph‐ and Dione‐COF films. As both local and long‐range interactions can affect exciton relaxation dynamics,^[27]^ the observed differences likely originate from variations of internal and external domain sizes and density of grain boundaries of the Aza‐Ph‐COF in comparison to Aza‐Naph‐COF and Dione‐COF.

## Conclusion

In summary, a one‐pot, three‐component methodology for the simultaneous construction and functionalization of covalent organic framework bulk and films is presented. Here, fragmental building block counterparts incorporated into a COF reaction mixture react at specific sites to form larger COF building elements, which are eventually fully integrated into the framework material. The construction of the building elements and the COF formation occur in one pot and on a similar time scale. The combination of pyrene dione diboronic acid, diamine, and hexahydroxytriphenylene building blocks in the reaction mixture gives rise to a series of pyrene‐fused azaacene boronate ester COFs, termed the Aza‐COF series, consisting of highly ordered Aza‐Ph, Aza‐Naph‐, and Aza‐2Ph‐COFs. Upon addition of reactive amines to the precursor solution, the aromatic system of the pyrene dione building block is quantitatively extended, resulting in fully overlapped or antiparallel AA stacking arrangements for the resulting COFs.

Powder X‐ray diffraction analysis of the synthesized COFs, coupled with structure simulation and calculations of lattice energies, suggests that the linearly extended dione moieties in Aza‐Ph‐COF promote a slip‐stacked AA alignment. In contrast, the steric hindrance from the phenyl groups in Aza‐2Ph‐COF leads to an antiparallel arrangement of the azaacenes in successive layers.

Moreover, the three‐component synthesis enables the fabrication of highly crystalline and oriented Aza‐COF thin films. The light absorption onset of these films gradually shifts to longer wavelengths with extending π‐conjugation. Photoluminescence measurements show similar trends with a weak emission profile. Additionally, transient absorption measurements of the thin films reveal temporal dynamics of excited states within the Aza‐COFs, accompanied by a recovery of the ground‐state bleaching at longer probe delays. Overall, this approach represents a significant addition to the toolkit of COF bulk and film synthesis methodologies, broadening the scope of COF functionalization.

## Conflict of interest

The authors declare no conflict of interest.

1

## Supporting information

As a service to our authors and readers, this journal provides supporting information supplied by the authors. Such materials are peer reviewed and may be re‐organized for online delivery, but are not copy‐edited or typeset. Technical support issues arising from supporting information (other than missing files) should be addressed to the authors.

Supporting Information

## Data Availability

The data that support the findings of this study are available from the corresponding author upon reasonable request.
