# Supplementary material for: Building Blocks and COFs Formed in Concert—Three‐Component Synthesis of Pyrene‐Fused Azaacene Covalent Organic Framework in the Bulk and as Films
Source: Angew Chem Int Ed Engl. 2023 Jun 13;62(30):e202302872. doi: 10.1002/anie.202302872 (PMC10952658; doi:10.1002/anie.202302872)
Supplement: Supplementary file 1 — Supporting Information [file ANIE-62-0-s001.pdf]

Angewandte Chemie
Eine Zeitschrift der Gesellschaft Deutscher Chemiker

Supporting Information

Building Blocks and COFs Formed *in Concert*—Three-Component Synthesis of Pyrene-Fused Azaacene Covalent Organic Framework in the Bulk and as Films

L. Frey, O. Oliveira, A. Sharma, R. Guntermann, S. P. S. Fernandes, K. M. Cid-Seara, H. Abbay, H. Thornes, J. Rocha, M. Döblinger, T. Kowalczyk, A. Rao, L. M. Salonen, D. D. Medina**

Supporting Information

Building Blocks and COFs Formed *in Concert*----Three-Component Synthesis of Pyrene-Fused Azaacene Covalent Organic Framework in the Bulk and as Films

Laura Frey,[a]≠ Orlando Oliveira,[c,d]≠ Ashish Sharma,[e] Roman Guntermann,[a] Soraia P. S. Fernandes,[c,f] Krystal M. Cid-Seara,[c,g] Hosanna Abbay,[h] Henry Thornes,[h] João Rocha,[d] Markus Döblinger,[a] Tim Kowalczyk,[h] Akshay Rao,[e] Laura M. Salonen,*[b,c] and Dana D. Medina*[a]

[a] L. Frey, R. Guntermann, Dr. M. Döblinger, Dr. D. D. Medina
Department of Chemistry and Center for Nanoscience (CeNS)
Ludwig-Maximilians-University
Butenandtstraße 11 (E), 81377 Munich, Germany
E-mail: dana.medina@cup.lmu.de

[b] Dr. L. M. Salonen
CINBIO, Universidade de Vigo
Department of Organic Chemistry,
36310 Vigo, Spain
E-mail: lauramaria.salonen@uvigo.es

[c] O. Oliveira, S. P. S. Fernandes, K. M. Cid-Seara, Dr. L. M. Salonen
International Iberian Nanotechnology (INL)
Avenida Mestre José Veiga, 4715-330 Braga, Portugal

[d] O. Oliveira, Prof. J. Rocha
CICECO – Aveiro Institute of Materials, University of Aveiro
3810-193, Aveiro, Portugal

[e] Dr. A. Sharma, Prof. Dr. A. Rao
Cavendish Laboratory, University of Cambridge
19 JJ Thomson Avenue, Cambridge CB3 0HE, UK

[f] S. P. S. Fernandes
Associate Laboratory for Green Chemistry-Network of Chemistry and Technology (LAQV-REQUIMTE)
University of Aveiro, Campus
Universitário de Santiago, 3810-193 Aveiro, Portugal

[g] K. M. Cid-Seara
Department of Inorganic Chemistry
University of Vigo
Campus Universitário, As Lagoas-Marcosende, 36310 Vigo, Spain

[h] H. Abbay, H. Thornes, Dr. T. Kowalczyk
Department of Chemistry and Advanced Materials Science and Engineering Center (AMSEC)
Western Washington University
516 High Street, Bellingham, WA 98225, USA

≠These authors contributed equally.

Table of contents

1. Materials and Methods 3

2. Synthetic Procedures 6

2.1. Synthesis of boronic acid-bearing pyrene-4,5-dione building block 6

2.2. Synthesis of Dione-COF 10

2.3. Three-component synthesis of Aza-COFs 10

3. NMR Spectra 12

4. Fourier-transform (FT-IR) spectra 25

5. Structural analysis 26

6. N_2 Physisorption 50

7. Transmission electron microscopy (TEM) 51

8. Thermogravimetric Analysis (TGA) 52

9. Thin film analysis 53

10. Photophysical properties 54

11. Transient absorption measurements 54

12. References 56

1. Materials and Methods

General: Commercial reagents and solvents were obtained from diverse suppliers and used as received, with an exception of pyrene, which was recrystallized prior to use from a mixture of CH_2Cl_2/hexane. Reaction progresses were monitored by analytical thin-layer chromatography (TLC) on ALUGRAM® Xtra SIL G/UV_{254} aluminium sheets from Macherey-Nagel. TLC plates were rendered visible by exposure to ultraviolet light. All purifications were carried out under flash-chromatographic conditions on silica gel (60 Å, 40–63 μm, 230–400 mesh, Macherey Nagel).

Nuclear magnetic resonance (NMR): The spectra were recorded using Bruker AV 400 and AV 400 TR spectrometers. Chemical shifts are expressed in parts per million (δ scale) and are calibrated using residual (undeuterated) solvent peaks as an internal reference (^{1}H NMR: $CDCl_3$: 7.26, DMSO-d_6: 2.50, Mesitylene-d_{12}: 2.08; ^{13}C NMR: $CDCl_3$: 77.16, DMSO-d_6: 39.52). Data for ^{1}H NMR spectra are reported in the following way: chemical shift (δ ppm) (multiplicity, coupling constant/ Hz, integration). Multiplicities are reported as follows: s = singlet, d = doublet, t = triplet, q = quartet, m = multiplet, or combinations thereof. **Solid-state ^{1}H-^{13}C cross-polarization magic angle spinning (CP-MAS) NMR** spectra were recorded on a Bruker Avance III-500 spectrometer (11.74 T) at 125.79 MHz, using 4 mm diameter ZrO_2 rotors and a spinning frequency of 12 kHz.

Powder X-ray diffraction (PXRD): The patterns were obtained on a Bruker D8 Advance diffractometer in Bragg-Brentano geometry equipped with a Cu K_α source (0.1 mm divergence slit, knife edge air scatter screen) and a LynxEye detector. K_β radiation was attenuated with a 0.0125 mm Ni filter.

Modeling of COF structures: Unit cells of the Aza-COFs were constructed according to the molecular symmetry of the building blocks and the expected connectivity. To build the crystal models the highest possible symmetry was applied using the Forcite module implemented in the Accelrys Materials Studio software package. The unit cells obtained were refined regarding their cell parameters using the Pawley method employing the Reflex module of the Materials Studio software.

Computational Methods: Initial geometries were prepared from the force field obtained CIFs via application of symmetry operations in Avogadro to fill the atoms (everything but hydrogen) of the unit cell. Hydrogen atoms were added via automation based on local bonding and adjusted to account for covalent bonding at unit cell boundaries. An initial constrained optimization corrected the placement of hydrogen atoms without modifying the positions of any heavier atoms. The resulting structures were then optimized at the DFTB level as follows. Self-consistent charge density functional tight-binding (SCC-DFTB) energies and gradients were calculated using matsci-0-3 Slater-Koster parameters in DFTB+. Geometry optimizations included lattice parameter optimization with periodic boundary conditions evaluated at the Γ point. An empirical Lennard-Jones dispersion correction was employed with standard UFF parameters.

2D grazing-incidence wide angle X-ray scattering (GIWAXS): The data was recorded with an Anton Paar SAXSpoint 2.0 system equipped with a Primux 100 micro Cu Kα source and a Dectris EIGER R 1M 2D detector. The COF films were positioned at a sample-detector distance of 140 mm and were measured with an incidence angle of 0.2°.

Nitrogen sorption isotherms: The isotherms were recorded with Quantachrome Autosorb 1 and Autosorb iQ instruments at 77 K. The samples were outgassed for 24 h at 120 °C under high vacuum prior to the measurements. Surface areas of the obtained powders were estimated by the multipoint Brunauer–Emmett–Teller (BET) method using ASIQwin™ software. Pore size distributions were calculated using quenched solid density functional theory (QSDFT) model with a carbon kernel for cylindrical pores (N_2 at 77 K on carbon, adsorption branch).

Fourier-transform infrared spectroscopy (FT-IR): The spectra were recorded on a Bruker VERTEX 70 FT-IR instrument using a liquid nitrogen-cooled MCT detector and a germanium ATR crystal. The IR data is background corrected and reported in frequency of adsorption (cm^{-1}).

Thermogravimetric analysis (TGA): The measurements were performed on a Netzsch Jupiter ST 499 C instrument equipped with a Netzsch TASC 414/4 controller. The samples were heated from room temperature to 900 °C under a synthetic air flow (25 ml min^{-1}) at a heating rate of 10 K min^{-1}.

Scanning electron microscopy (SEM): The images were recorded with a FEI Helios NanoLab G3 UC scanning electron microscope equipped with a field emission gun operated at 3–5 kV.

Transmission electron microscopy (TEM) was performed on a FEI Titan Themis instrument equipped with a field emission gun operated at 300 kV.

UV-VIS-NIR: The spectra were recorded on a Perkin-Elmer Lambda 1050 spectrometer equipped with a 150 mm InGaAs integrating sphere. **Diffuse reflectance spectra** were collected with a Praying Mantis (Harrick) accessory and were referenced to barium sulfate powder as white standard. The specular reflection of the sample surface was removed from the signal using apertures that allow only light scattered at angles > 20° to pass.

Photoluminescence (PL): The measurements were performed using a PicoQuant FluoTime300 time-correlated single photon counting setup. The steady-state spectrum was recorded using a 378 nm laser (PicoQuant LDH-P-C-375).

Transient absorption measurements: The transient absorption (TA) measurements were performed using in a custom setup, where the laser source was a Ti:sapphire amplifier system (Spectra-Physics Solstice) operating at a frequency of 1 kHz. A part of the 1 kHz pulse is frequency doubled using a BBO crystal to generate the pump beam (400 nm). Another part of the output from the Ti:sapphire system is focussed on a CaF_2 crystal to generate broadband probe beams in the visible region. The pump and probe beams are overlapped on the sample and are focused into an imaging spectrometer (Andor, Shamrock SR 303i). The beams are detected using a pair of linear image sensors (Hamamatsu, G11608) driven and read out at the full laser repetition rate by a custom-built board from Stresing Entwicklungsburo. A chopper

is used to have pump-on and pump-off periods during all measurements, which enables the system software to calculate differential transmission, $\Delta T/T$. The samples used for the TA experiments were encapsulated films coated on sapphire substrates (Ossila) to minimize heating effects.

High-resolution electrospray ionization (ESI) mass spectra (MS) were recorded with a Thermo Finnigan MAT 95 instrument.

Elemental microanalysis measurements were recorded using an Elementar vario EL instrument.

2. Synthetic Procedures

2.1. Synthesis of boronic acid-bearing pyrene-4,5-dione building block

Starting point of the synthetic route of boronic acid-bearing pyrene-4,5-dione building block is the selective ruthenium-catalyzed oxidation of pyrene in the 4 and 5 positions to afford pyrene-4,5-dione (**1**).[1] The following protection of ketone groups using ethylene glycol gave **2** in 43% yield.[2] Next, iridium-catalyzed C–H borylation takes place at the 2,7-positions to afford **3** in 49% yield.[2] Finally, deprotection of both protecting groups at room temperature with $NaIO_4$ under acidic conditions gave boronic acid-bearing pyrene-4,5-dione building block in 88% yield.[2,3]

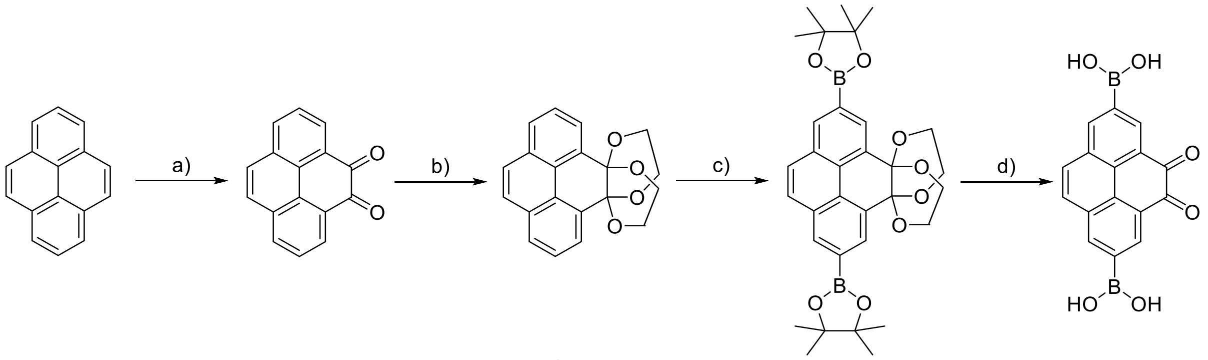

1 **2** **3**

Scheme S1. Synthetic route of boronic acid-bearing pyrene-4,5-dione building block. a) CH_2Cl_2, THF, $RuCl_3$•$2H_2O$, *N*-methylimidazole, H_2O, $NaIO_4$, 2.5 h, 50%; b) ethylene glycol, PTSA, toluene, 125 °C, 20 h, 43%; c) bis(pinacolato)diboron, $[Ir(OMe)COD]_2$, 4,4'-di-*tert*-butyl-2,2'-bipyridine, MTBE, 80 °C, 20 h, 49%; d) $NaIO_4$, TFA, THF, H_2O, air, RT, 3 d, 88%.

(7-Borono-4,5-dioxo-pyren-2-yl)boronic acid (Pyrene dione building block)[3]

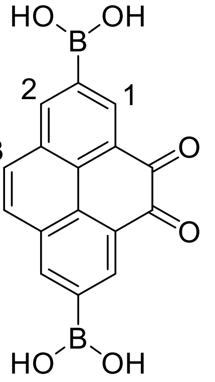

In a round-bottom flask, **3** (522 mg, 0.91 mmol, 1 equiv.) was dissolved in a mixture of THF/H_2O (41/12 mL). $NaIO_4$ (1.65 g, 7.71 mmol, 8.5 equiv.) was added, and the mixture was stirred at room temperature for 30 min. Then, TFA (85 mL) was added dropwise and the reaction mixture was stirred for 3 days at room temperature while bubbling air through the solution. An orange solid precipitated and the suspension was diluted with CH_2Cl_2 and H_2O. The aqueous phase was extracted with CH_2Cl_2, and the organic phase was filtered. The collected solid was combined with the aqueous phase and centrifuged (15 min, 4400 rpm). The

orange solid was suspended in water and the centrifugation was repeated. This last washing step was repeated four times in total. The solid was dried under high vacuum to give pyrene dione building block (255 mg, 88%) as an orange solid.

^{1}**H NMR** (400 MHz, $(CD_3)_2SO+D_2O$): δ = 8.72 (d, J = 1.6 Hz, 2H; H–C(1)), 8.68 (d, J = 1.2 Hz, 2H; H–C(2)), 8.01 (s, 2H; H–C(3)).

Pyrene-4,5-dione (1)[1]

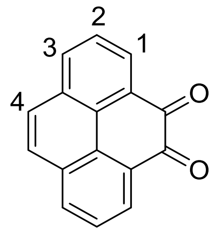

In a round-bottom flask, pyrene crystals (9.7 g, 47.9 mmol, 1 equiv.) were dissolved in a mixture of CH_2Cl_2 and THF (240/240 mL). To the yellow solution $RuCl_3$•$2H_2O$ (0.99 g, 4.79 mmol, 0.1 equiv.), *N*-methylimidazole (190.5 µL, 2.39 mmol, 0.05 equiv.), and water (240 mL) were added. Then, over a period of 20 minutes, sodium periodate (46.1 g, 215.4 mmol, 4.5 equiv.) was added in small portions, and the mixture was stirred at RT for 2.5 h. Organic solvents were removed under reduced pressure, and the aqueous phase was extracted with CH_2Cl_2. The collected organic phase was washed with water, dried with anhydrous Na_2SO_4, and concentrated under reduced pressure to afford a dark orange solid. Purification by column chromatography (SiO_2; CH_2Cl_2) gave **1** (5.5 g, 23.7 mmol, 50%) as bright-orange solid.

$\boldsymbol{R_f}$ = 0.3 (CH_2Cl_2).

^{1}**H NMR** (400 MHz, $CDCl_3$): δ = 8.44 (dd, J = 7.4 Hz, J = 1.3 Hz, 2H, H–C(1) or H–C(3)), 8.13 (dd, J = 7.9 Hz, J = 1.3 Hz, 2H, H–C(1) or H–C(3)), 7.80 (s, 2H, H–C(4)), 7.72 (dd, J = 7.9 Hz, J = 7.5 Hz, 2H, H–C(2)).

Pyrene-4,5-di(ethyleneglycol)ketal (2)[2]

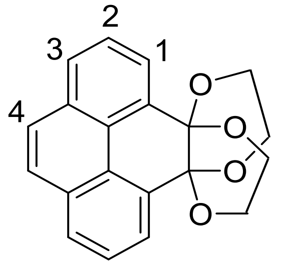

To a suspension of pyrene-4,5-dione (**1**) (2.99 g, 12.9 mmol, 1 equiv.) in toluene (150 mL) were added ethylene glycol (90 mL, 1.61 mol, 125 equiv.) and *p*-toluenesulfonic acid (1.11 g, 5.81 mmol, 0.45 equiv.). The obtained orange reaction mixture was refluxed at 125 °C for 20 h. After the reaction mixture was cooled down, toluene was removed under reduced pressure. Then, 200 mL of water was added and a light brown solid precipitated, which was collected by

filtration and washed with water. Purification by flash column chromatography (SiO_2; cyclohexane/ethyl acetate 9:1) gave **2** (1.80 g, 5.54 mmol, 43%) as white solid.

***R*f** = 0.29 (cyclohexane/EtOAc 9:1).

^{1}H NMR (400 MHz, CD_2Cl_2): δ = 7.94 (dd, *J* = 7.6 Hz, *J* = 1.2 Hz, 4H; H–C(1) or H–C(3)), 7.80 (s, 2H; H–C(4)), 7.70 (t, *J* = 7.6 Hz, 2H; H–C(2)), 4.27 (br, 4H), 3.73 (br, 4H).

2,7-Bis(Bpin)-4,5-di(ethyleneglycol)ketal-pyrene (3)[2]

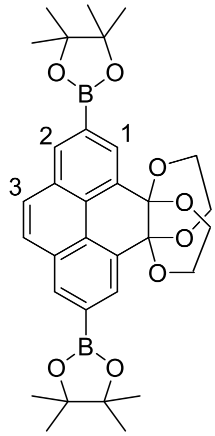

To a nitrogen-filled 100 mL pressure tube (Ace glass, bushing type back seal, 17.8 cm x 38.1 mm), **2** (0.89 g, 2.76 mmol, 1 equiv.), bis(pinacolato)diboron (1.41 g, 5.52 mmol, 2 equiv.), $[Ir(OMe)COD]_2$ (0.09 g, 0.14 mmol, 0.05 equiv.), 4,4'-di-*tert*-butyl-2,2'-bipyridine (0.07 g, 0.28 mmol, 0.1 equiv.), and *tert*-butyl methyl ether (18 mL) were added. The reaction mixture was stirred at 80 ºC for 20 h, cooled down and passed through a pad of silica using toluene as the eluent. The solvent was removed under reduced pressure and the product was purified by flash column chromatography (SiO_2; cyclohexane/ethyl acetate 9:1 → 8:2 → 7:3), as off-white solid (0.77 g, 0.95 mmol, 49%).

***R*f** = 0.2 (cyclohexane/EtOAc 9:1).

^{1}H NMR (400 MHz, CD_2Cl_2): δ = 8.36 (d, *J* = 1.2 Hz, 2H; H–C(1) or H–C(2)), 8.24 (d, *J* = 1.2 Hz, 2H; H–C(1) or H–C(2)), 7.81 (s, 2H; H–C(3)), 4.28 (br, 4H), 3.71 (br, 4H), 1.37 (s, 24H).

Aza-Ph model system

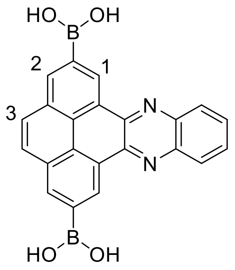

Boronic acid-bearing **pyrene dione building block** (12 mg, 0.04 mmol); *o*-phenylenediamine (4 mg, 0.04 mmol); DMSO (1 mL); brown **Aza-Ph** model system (9 mg, 0.02 mmol, 50%).

^{1}H NMR (400 MHz, $(CD_3)_2SO+D_2O$): δ= 9.86 (d, *J* = 1.2 Hz, 2H; H−C(1) or H−C(2)), 8.79 (d, *J* = 1.2 Hz, 2H; H−C(1) or H−C(2)), 8.41 (dd, J_1 = 3.4 Hz, J_2 = 6.5 Hz, 2H; H−Ph), 8.15 (s, 2H; H−3), 7.99 (dd, J_1 = 3.4 Hz, J_2 = 6.5 Hz, 2H; H−Ph). ^{13}C NMR (100 MHz, $(CD_3)_2SO+D_2O$): 143.4, 142.1, 135.9, 131.1, 130.9, 129.5, 129.5, 128.3, 127.8, 126.7 ppm. IR (ATR): 3448, 3415, 1662, 1610, 1520, 1468, 1416, 1352, 1275, 1174, 1140, 1099, 891, 806, 744, 669, 563, 523.

2.2. Synthesis of Dione-COF

Dione-COF was synthesized according to our previous report,[3] in a 10 mL ampoule (Wheaton, prescored, borosilicate, 19 x 107 mm) flushed with argon. Pyrene dione building block (30 mg, 0.10 mmol, 1 equiv.) and 2,3,6,7,10,11-hexahydroxytriphenylene (HHTP) hydrate (20 mg, 0.06 mmol, 0.64 equiv.) were added to a solvent mixture of 1:1 mesitylene and *n*-butanol (3.5 mL), previously degassed by three cycles of freeze-pump-thaw. The reaction mixture was sonicated for 5 min. The ampoule was immersed in a bath of liquid N_2, sealed under vacuum, and heated in the oven at 120 ºC for 7 days. The precipitate was collected by centrifugation, washed with anhydrous acetone four times, and dried under nitrogen at 90 ºC, to give 45 mg of Dione-COF as brown solid.

2.3. Three-component synthesis of Aza-COFs

General procedure

In a 6 mL culture tube under Ar, the boronic acid-bearing pyrene dione building block (8.57 mg, 28.6 µmol, 1 equiv.), 2,3,6,7,10,11-hexahydroxytriphenylene (HHTP) hydrate (5.71 mg, 17.1 µmol, 0.64 equiv.) and 2.1 equiv. of the respective amine (*o*-phenylenediamine (Ph): 6.49 mg, 0.06 mmol, 2.1 equiv.), 2,3-diaminonaphthalene (Naph): 9.50 mg, 0.06 mmol, 2.1 equiv.; or (1*R*,2*R*)-(+)-1,2-diphenylethylenediamine (2Ph): 6.49 mg, 0.06 mmol, 2.1 equiv.) were suspended in a 1:1 mixture of mesitylene and *n*-butanol (1 mL). The reaction mixture was heated at 120 ºC for 7 days. The precipitate was collected by filtration and washed four times with anhydrous acetone to afford Aza-Ph-COF, Aza-Naph-COF, and Aza-2Ph-COF with 100% conversion of the dione moieties, and yields of 48% (Aza-Ph-COF), 41% (Aza-Naph-COF), and 53% (Aza-2Ph-COF).

Aza-Ph-COF (for $C_{34}H_{14}B_2N_2O_4$): calculated C 76.17, found C 71.63; calculated H 2.63, found H 2.73; calculated N 5.23, found N 4.96. HR-ESI-MS: *m/z* (%): 421.1520 (100), 407.1366 (83), 393.1207 (65, $[M+H]^+$, calculated for $C_{22}H_{15}B_2N_2O_4^+$: 393.1212), 222.4763 (84); 323.0562 (100, $[HHTP-H]^-$, calculated for $C_{18}H_{11}O_6^-$: 323.0561).

Aza-Naph-COF (for $C_{38}H_{16}B_2N_2O_4$): calculated C 77.86, found C 78.08; calculated H 2.75, found H 3.49; calculated N 4.78, found N 4.62. HR-ESI-MS: *m/z* (%): 471.1677 (60), 425.2867 (63), 413.1451 (87), 399.1293 (43, $[M-BO_2]^+$, calculated for $C_{26}H_{16}BN_2O_2^+$: 399.1299), 222.4697 (100); 323.0562 (100, $[HHTP-H]^-$, calculated for $C_{18}H_{11}O_6^-$: 323.0561).

Aza-2Ph-COF (for $C_{42}H_{20}B_2N_2O_4$): calculated C 79.04, found C 78.64; calculated H 3.16, found H 4.10; calculated N 4.39, found N 4.04. HR-ESI-MS: *m/z* (%): 495.1687 (10, $[M+H]^+$, calculated for $C_{30}H_{21}B_2N_2O_4^+$: 495.1682), 421.1589 (62), 407.1372 (74, $[M-B_2O_4H_2]^+$, calculated for $C_{30}H_{19}N_2^+$: 407.1543), 299.1544 (100); 323.0561 (100, $[HHTP-H]^-$, calculated for $C_{18}H_{11}O_6^-$: 323.0561).

2.4. *Aza-COFs thin film synthesis*

General procedure

The thin films were synthesized using a bottom-up approach. A 100 mL Schott Duran glass bottle equipped with a Teflon holder and a horizontally positioned substrate was charged with the boronic acid-bearing pyrene dione building block (8.57 mg, 28.6 μmol, 1 equiv.), 2,3,6,7,10,11-hexahydroxytriphenylene (HHTP) hydrate (5.71 mg, 17.1 μmol, 0.64 equiv.) and 2.1 equiv. (0.06 mmol) of the respective amine (Ph, Naph, or 2Ph). Next, a mixture of mesitylene and *n*-butanol (2000 µL, 1:1 *v*:*v*) was added. The glass bottle was sealed and heated at 120 °C for 6 d. After cooling to room temperature, the substrate was recovered, and the COF film obtained was rinsed with anhydrous acetone and dried under a stream of nitrogen.

3. NMR Spectra

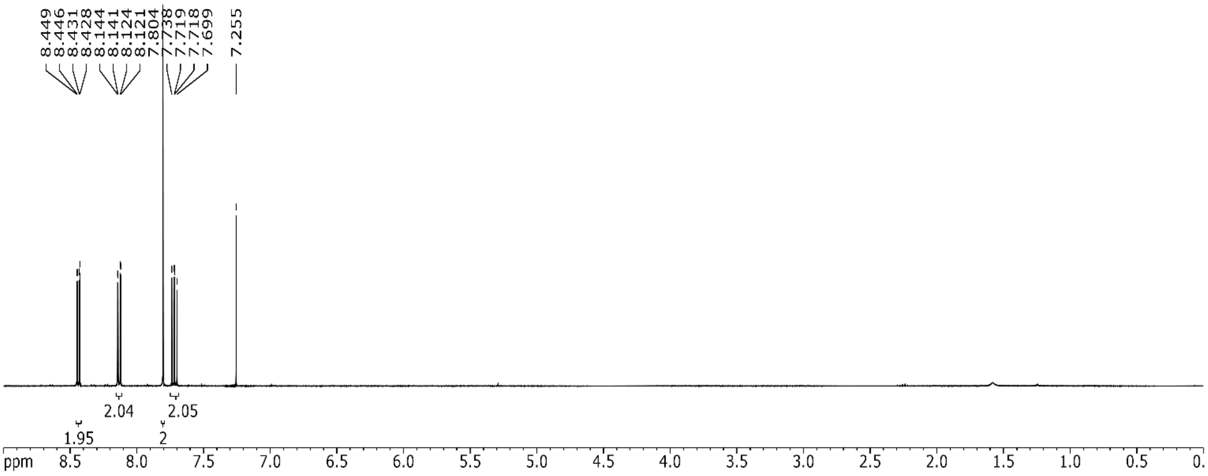

Figure S1. ^{1}H NMR spectrum (400 MHz, $CDCl_3$) of pyrene-4,5-dione (**1**).

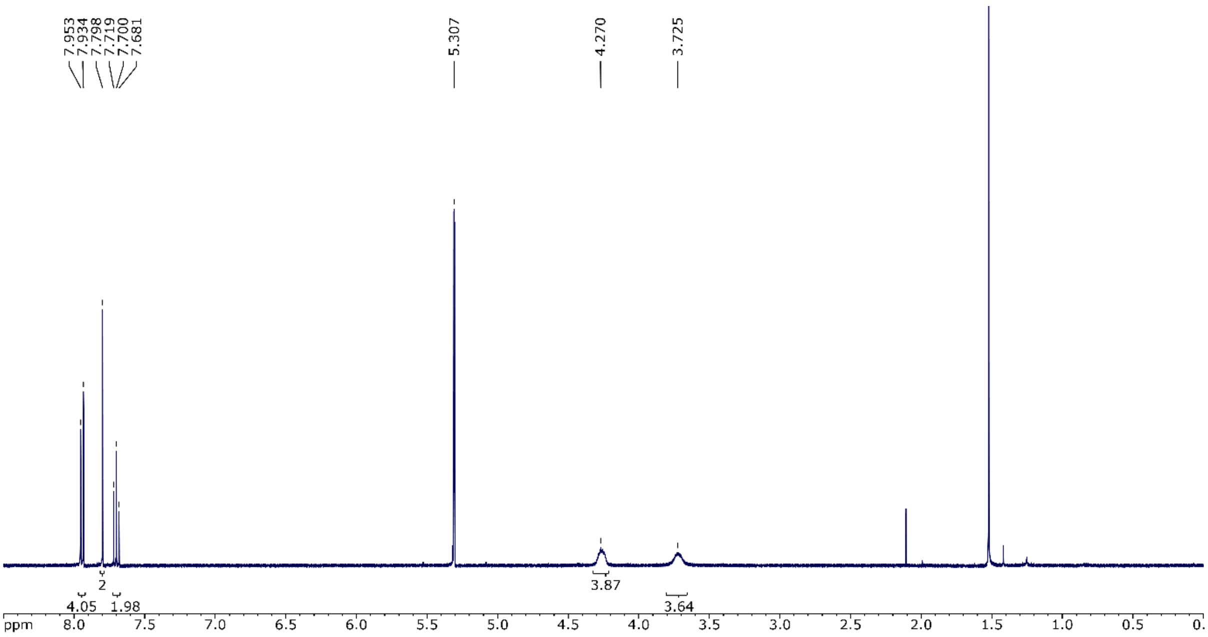

Figure S2. ^{1}H NMR spectrum (400 MHz, CD_2Cl_2) of pyrene-4,5-di(ethyleneglycol)ketal (**2**).

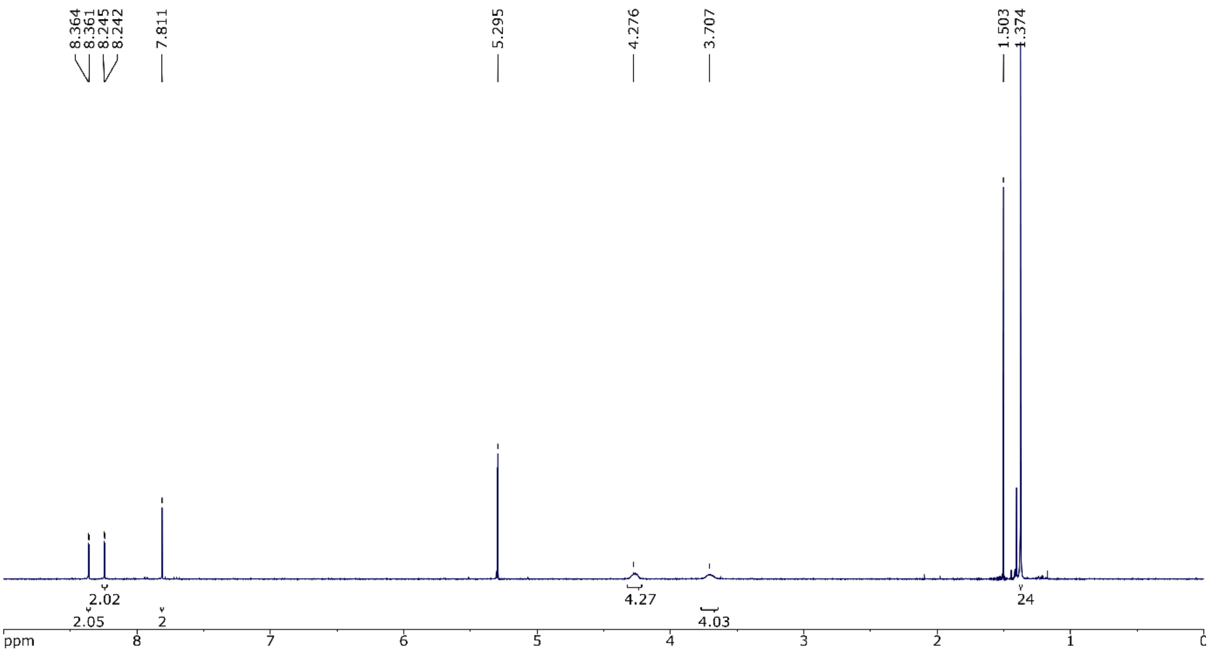

Figure S3. ^{1}H NMR spectrum (400 MHz, CD_2Cl_2) of 2,7-bis(Bpin)-4,5-di(ethyleneglycol)ketalpyrene (**3**).

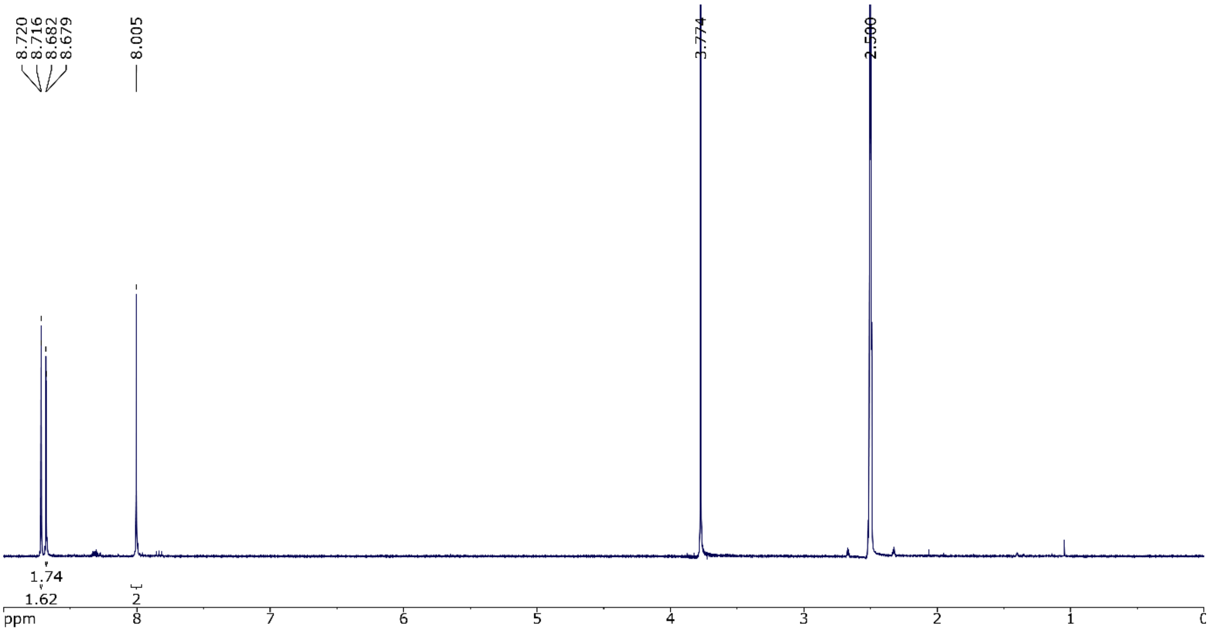

Figure S4. ^{1}H NMR spectrum (400 MHz, in $(CD_3)_2SO+D_2O$) of (7-borono-4,5-dioxo-pyren-2-yl)boronic acid (pyrene-4,5-dione building block **1**).

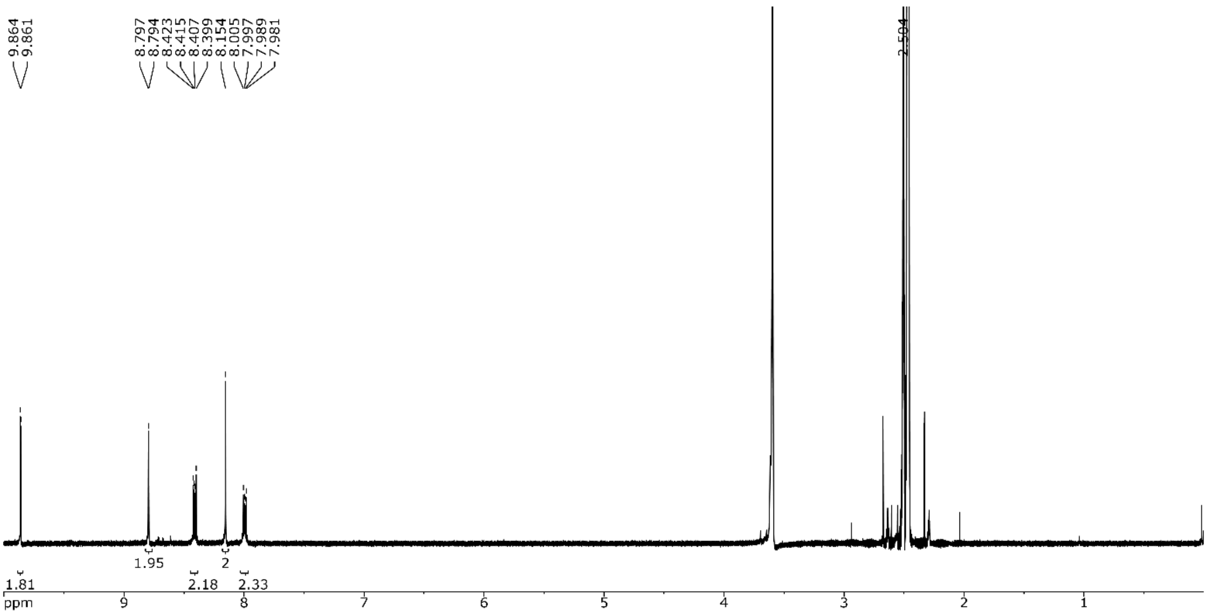

Figure S5. ^{1}H NMR spectrum of Aza-Ph model system measured at 400 MHz in $(CD_3)_2SO+D_2O$.

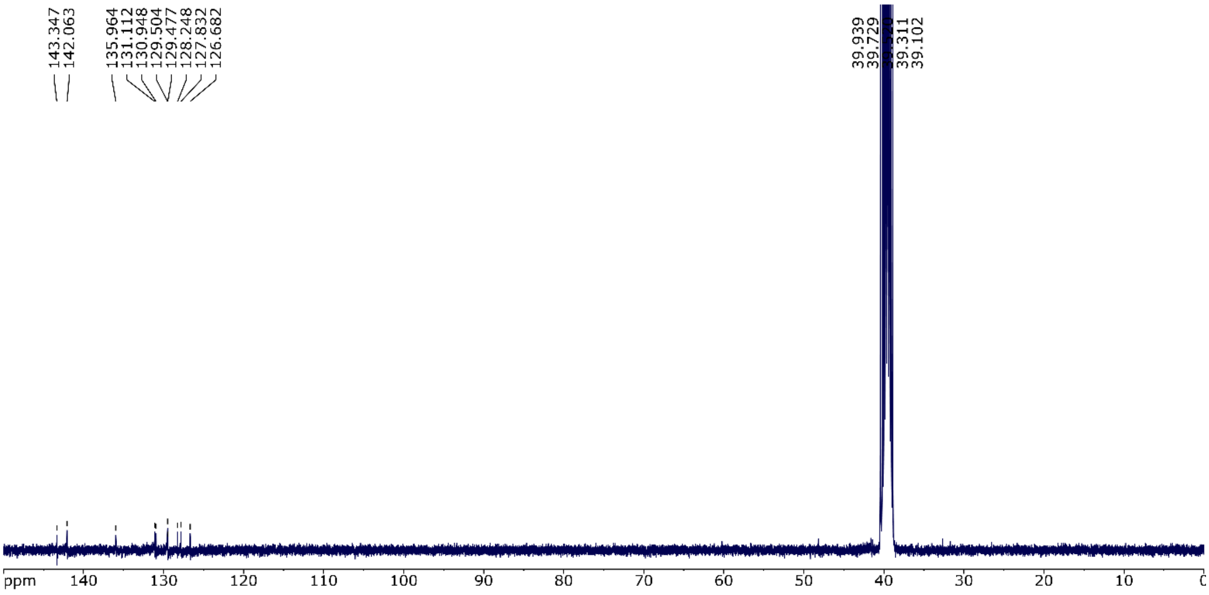

Figure S6. ^{13}C NMR spectrum of Aza-Ph model system measured at 100 MHz in $(CD_3)_2SO+D_2O$.

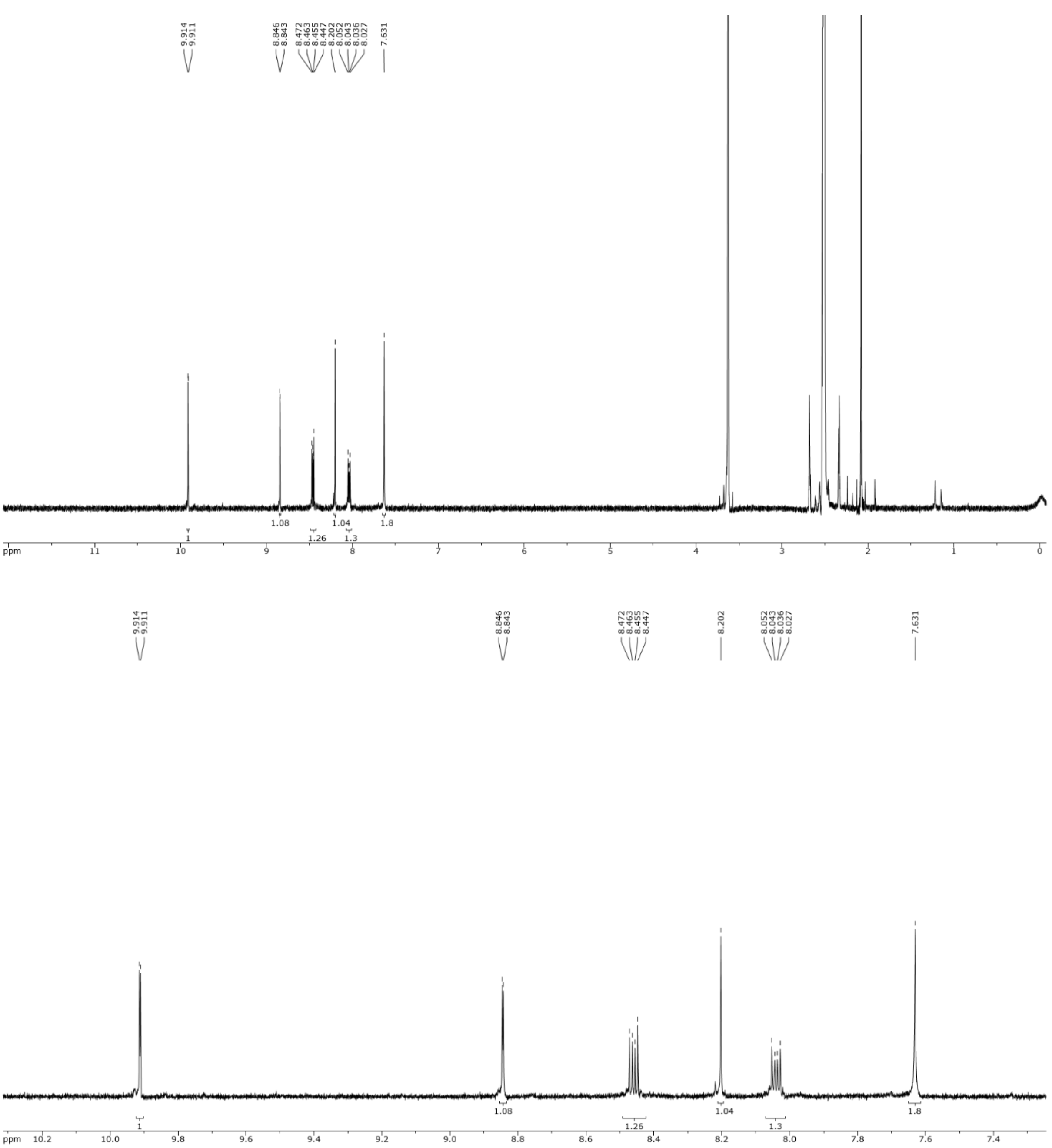

Figure S7. ^{1}H NMR spectrum (400 MHz, $(CD_3)_2SO+D_2O$) of Aza-Ph-COF (top) and aromatic zone zoom (bottom).

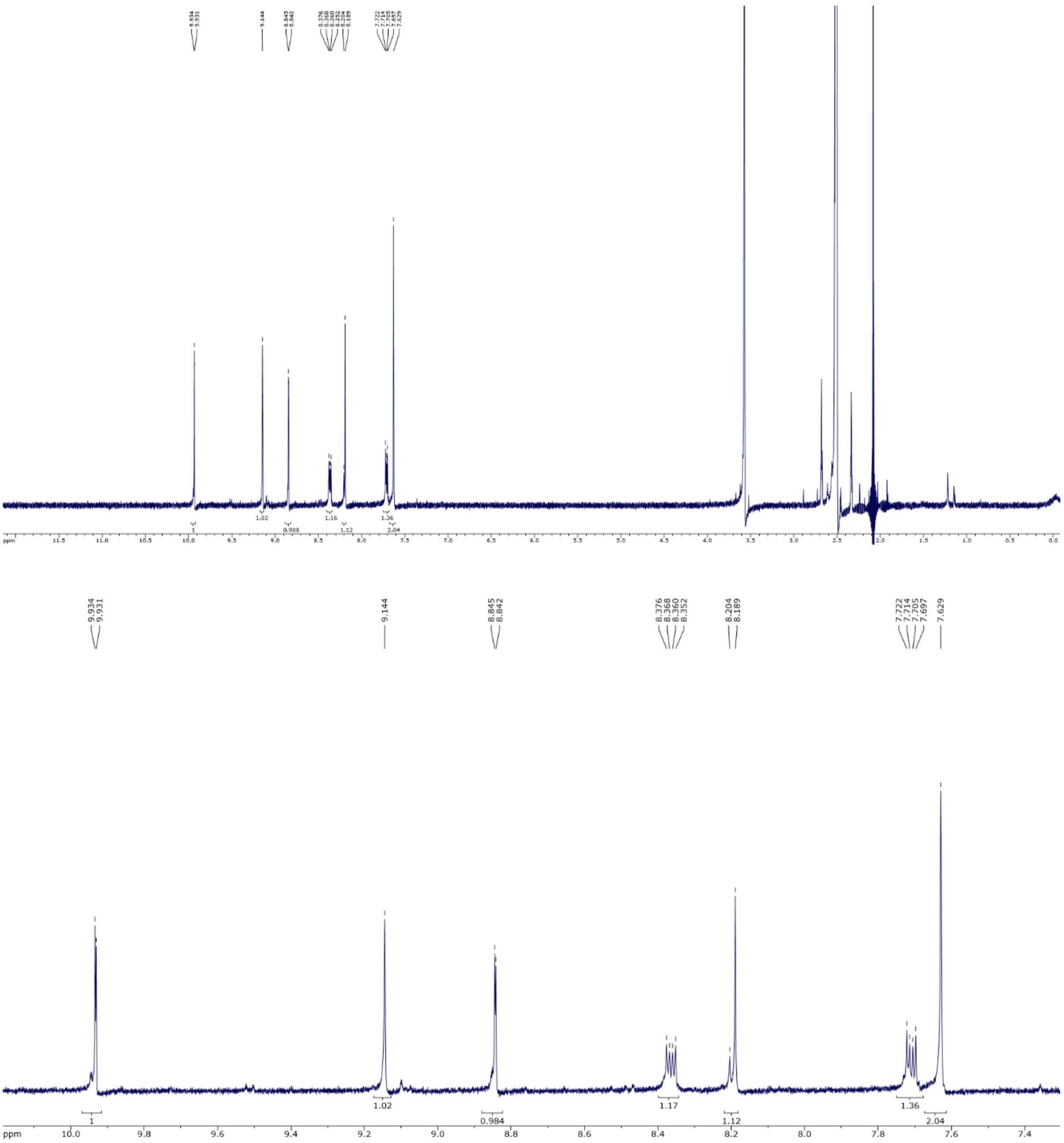

Figure S8. ^{1}H NMR spectrum (400 MHz, $(CD_3)_2SO+D_2O$) of Aza-Naph-COF (top) and aromatic zone zoom (bottom).

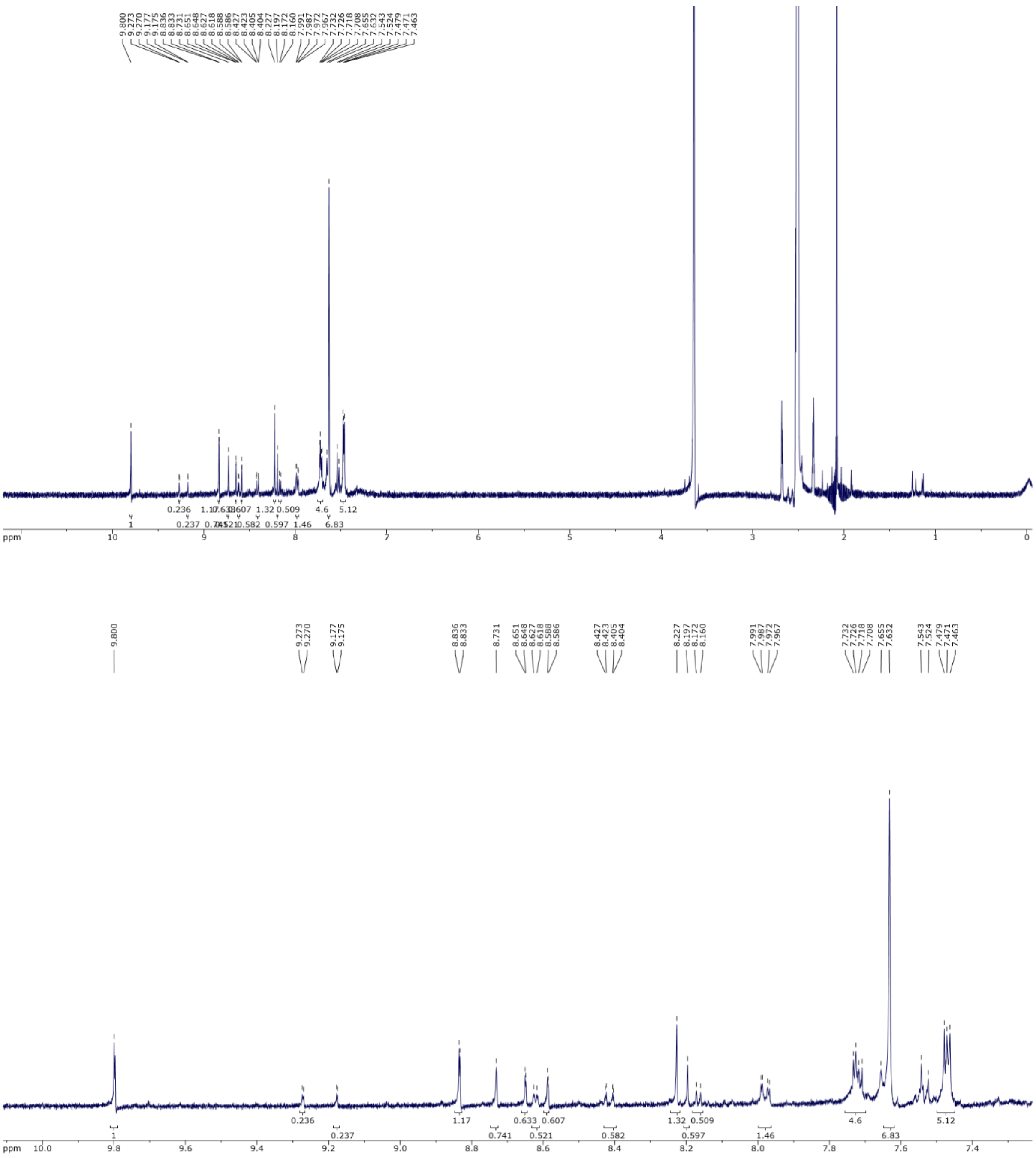

Figure S9. ^{1}H NMR spectrum (400 MHz, $(CD_3)_2SO$+D_2O) of Aza-2Ph-COF (top) and aromatic zone zoom (bottom).

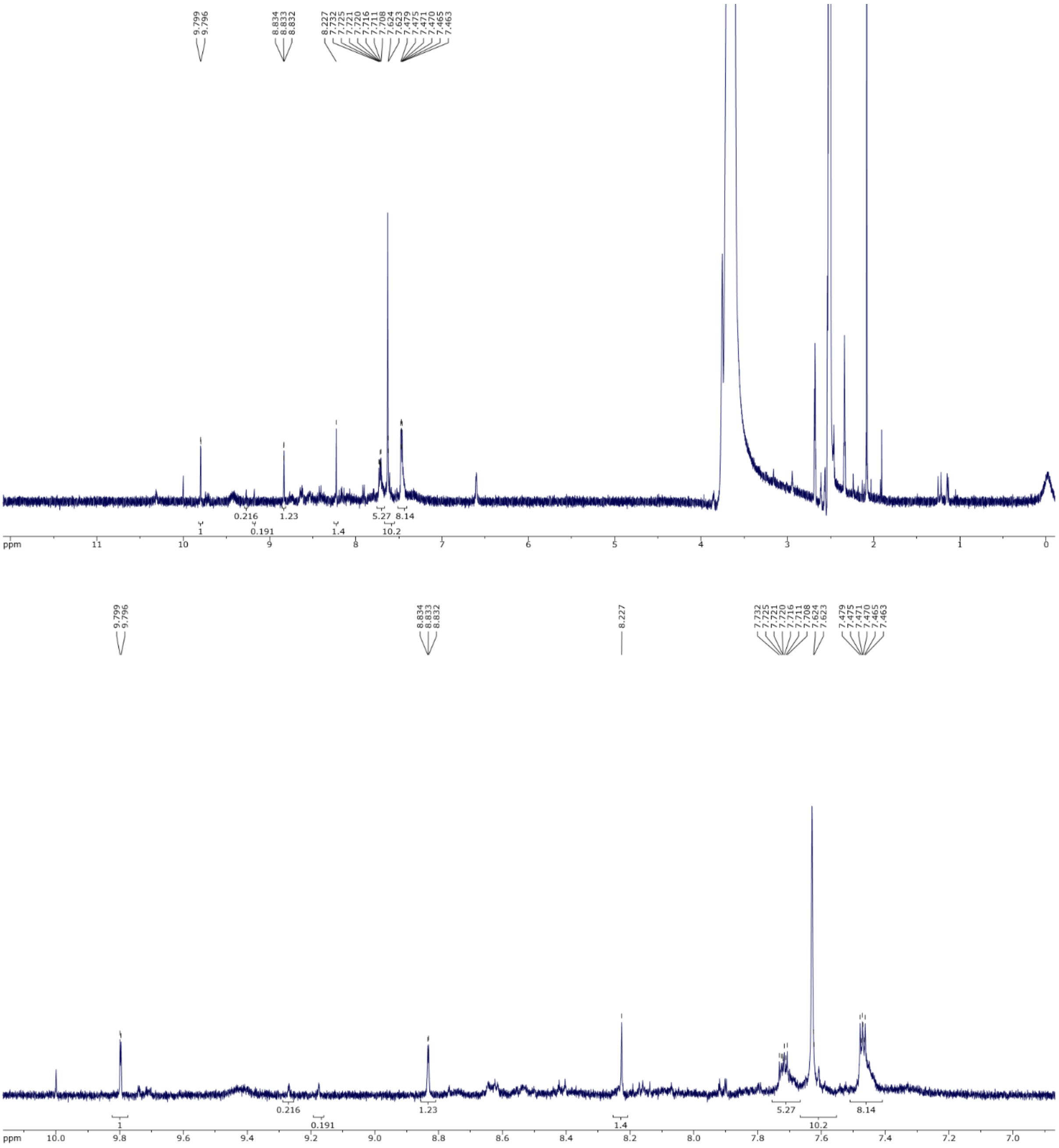

Figure S10. ^{1}H NMR spectrum (400 MHz, $(CD_3)_2SO+D_2O$) of Aza-2Ph-COF after 2 h of air bubbling through the NMR solution (top) and aromatic zone zoom (bottom).

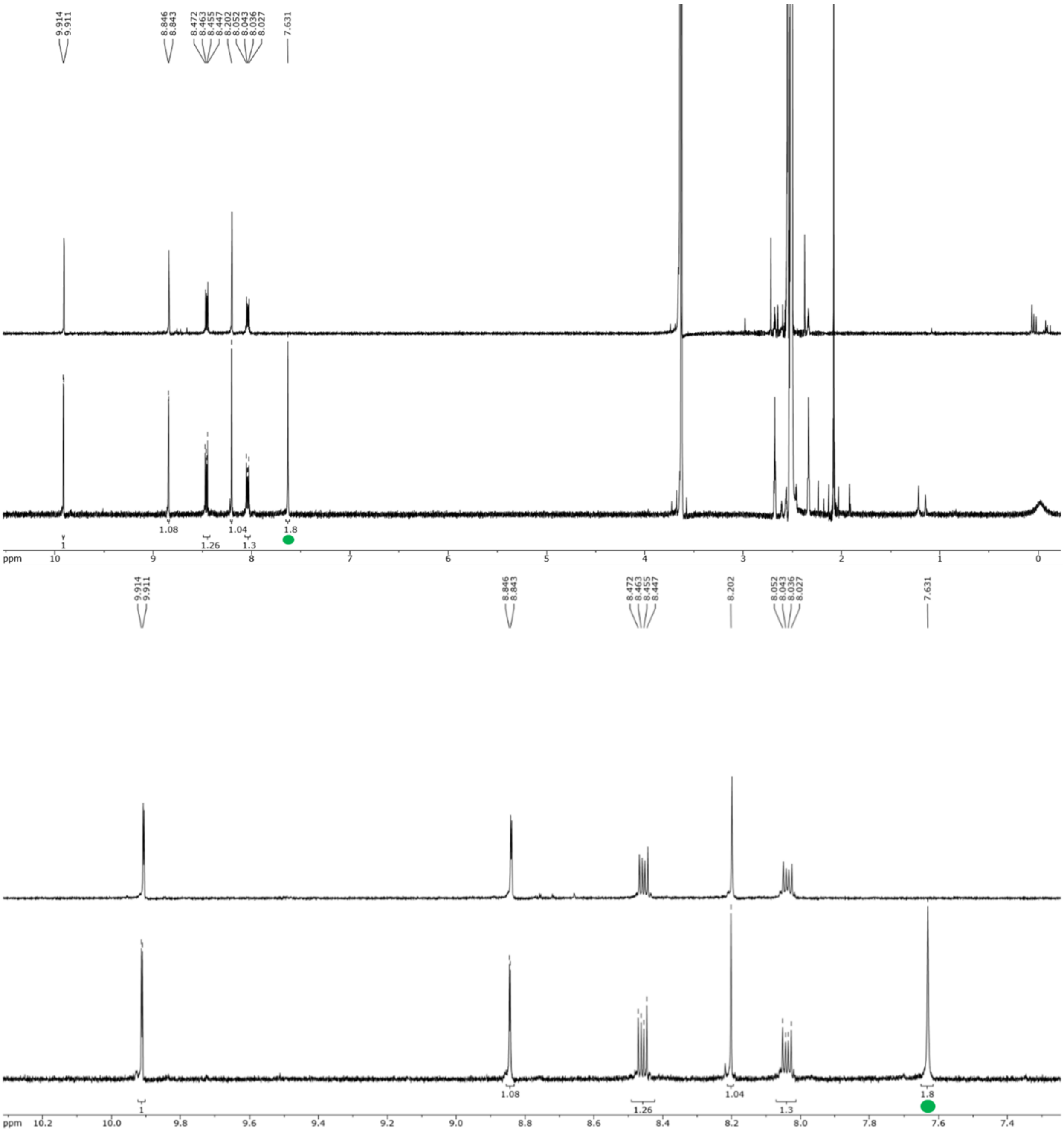

Figure S11. A comparison between the ^{1}H NMR spectra of the Aza-Ph model system (top) and Aza-Ph-COF (bottom), confirming the formation of the azaacene compound in the three-component synthesis. The signal stemming from the C–H protons of HHTP is marked with a green dot.

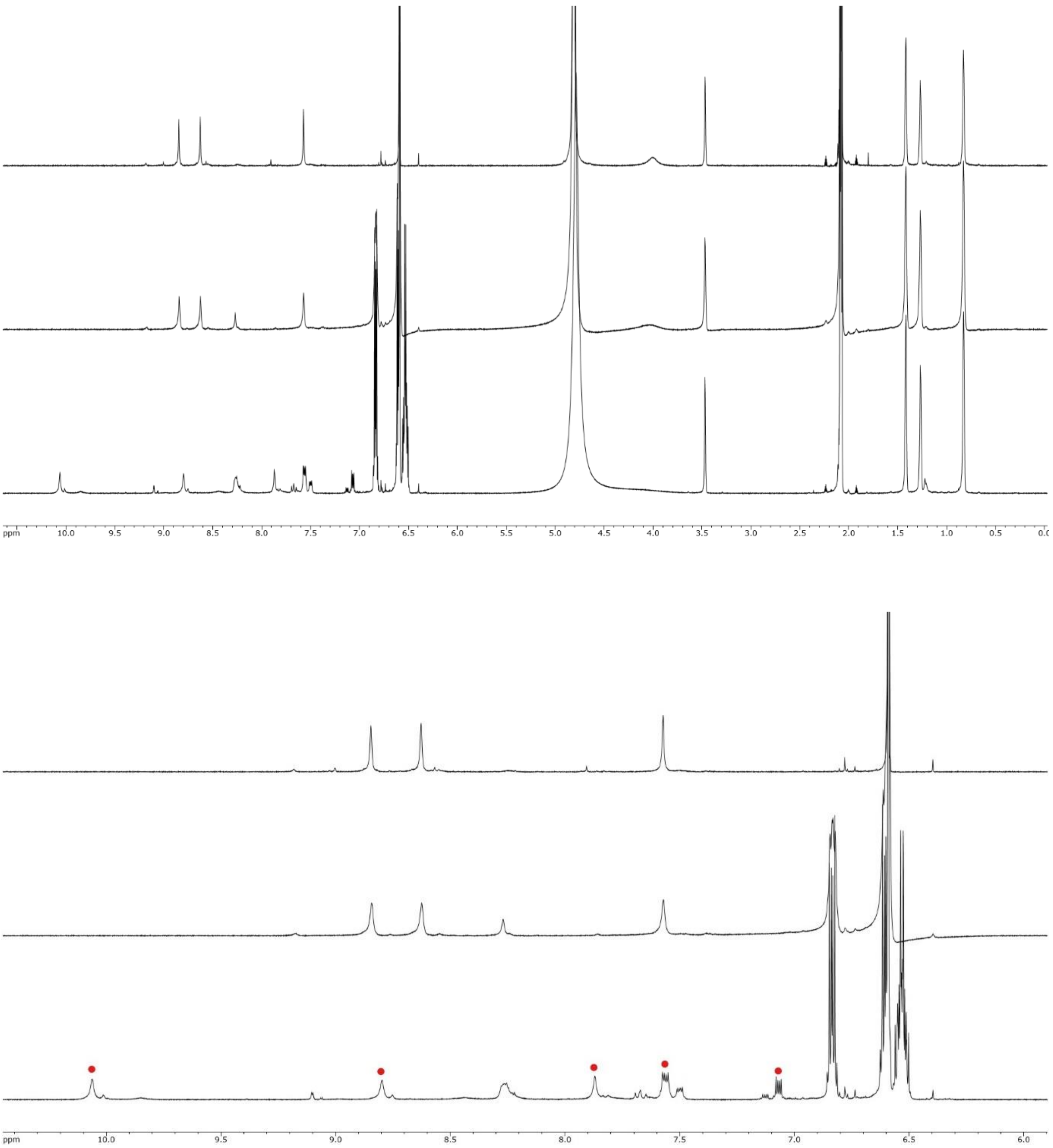

Figure S12. Overlay of the ^{1}H NMR spectra (400 MHz, deuterated mesitylene and deuterated *n*-butanol 1:1 (*v*/*v*), room temperature) of pyrene dione building block (top), after addition of catechol (2.2 equiv.; middle) and *o*-phenylenediamine (2.2 equiv.; bottom), respectively. Red dots indicate the azaacene peaks.

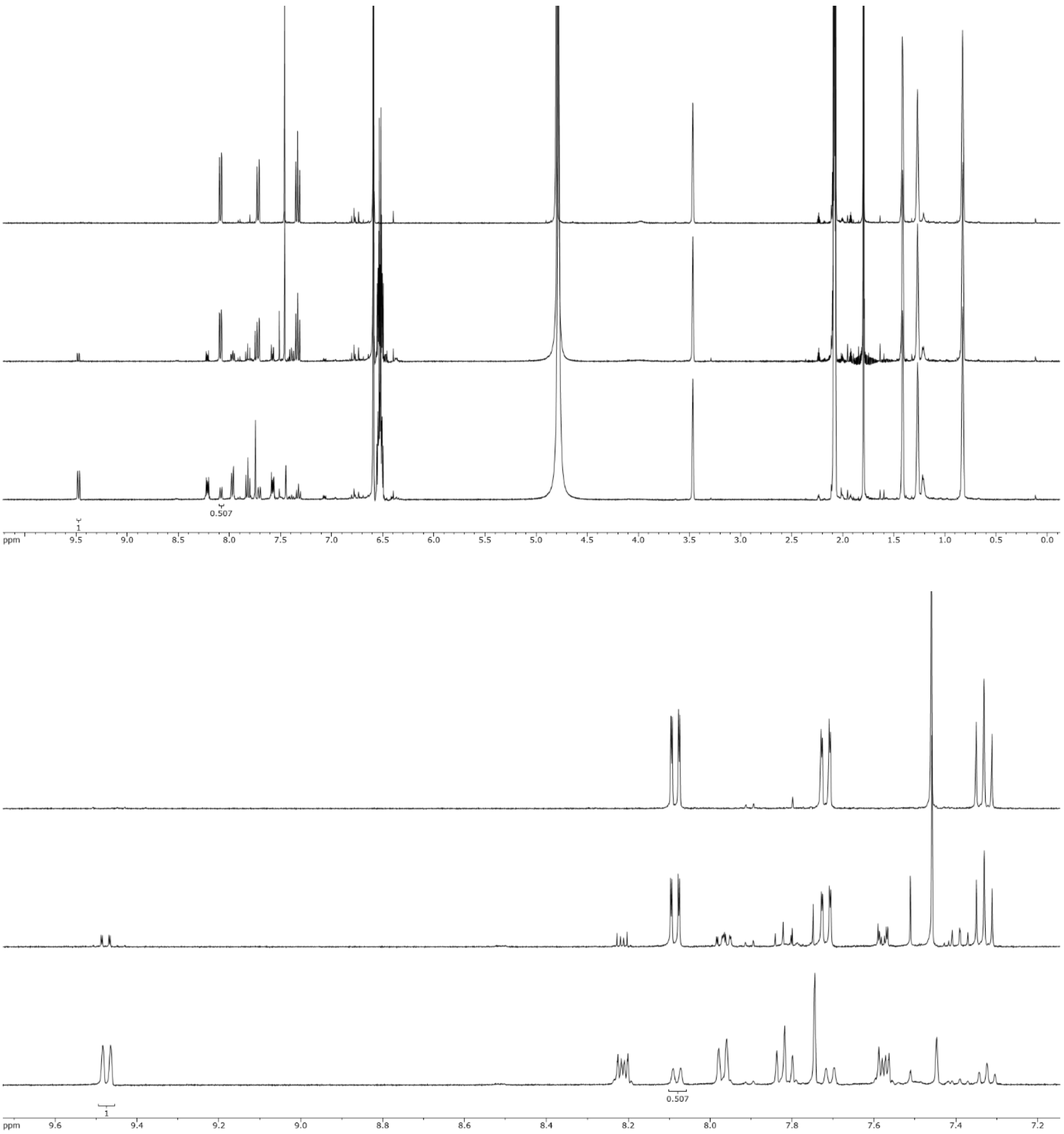

Figure S13. Overlay of the ^{1}H NMR spectra (400 MHz, deuterated mesitylene and deuterated *n*-butanol 1:1 (*v*/*v*), room temperature) of pyrene-4,5-dione (top), upon addition of *o*-phenylenediamine (2.2 equiv.; middle), and after 1 h of *o*-phenylenediamine addition (bottom), respectively.

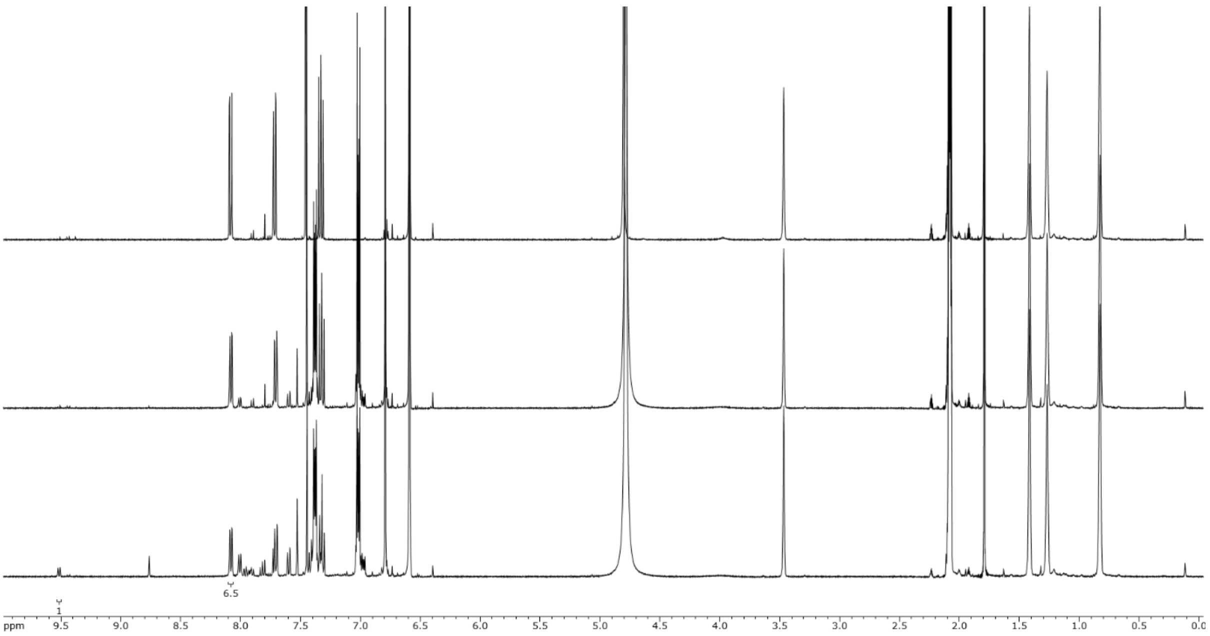

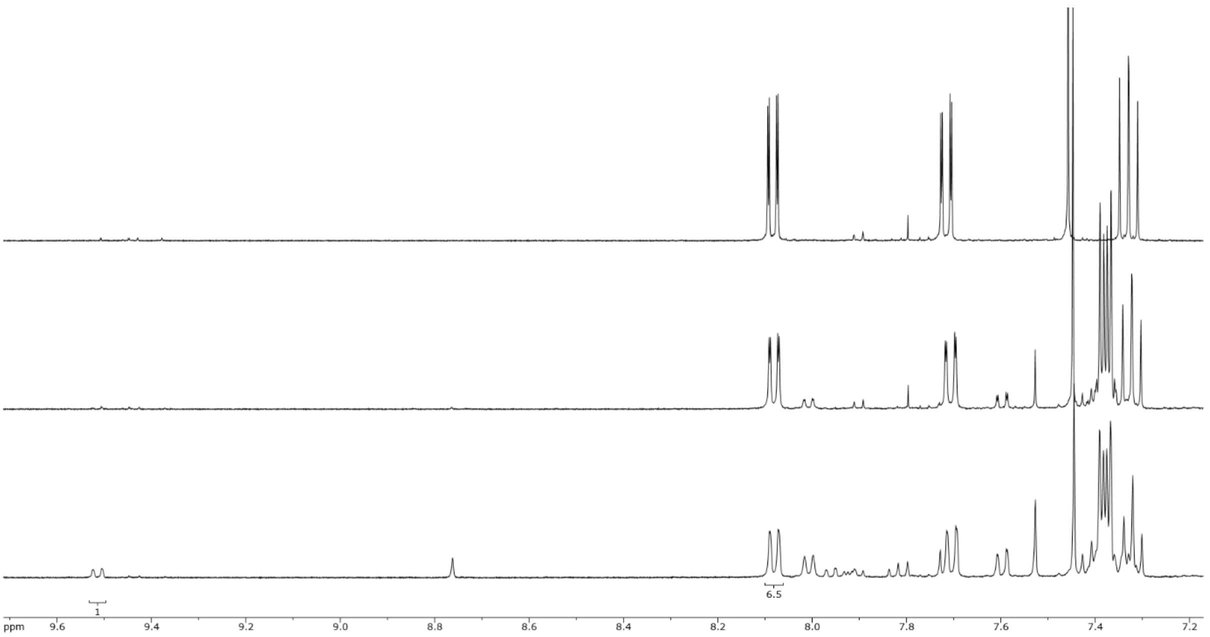

Figure S14. Overlay of the ^{1}H NMR spectra (400 MHz, deuterated mesitylene and deuterated *n*-butanol 1:1 (*v*/*v*), room temperature) of pyrene-4,5-dione (top), upon addition of 2,3-diaminonaphtalene (2.2 equiv.; middle), and after 1 h of 2,3-diaminonaphtalene addition (bottom), respectively.

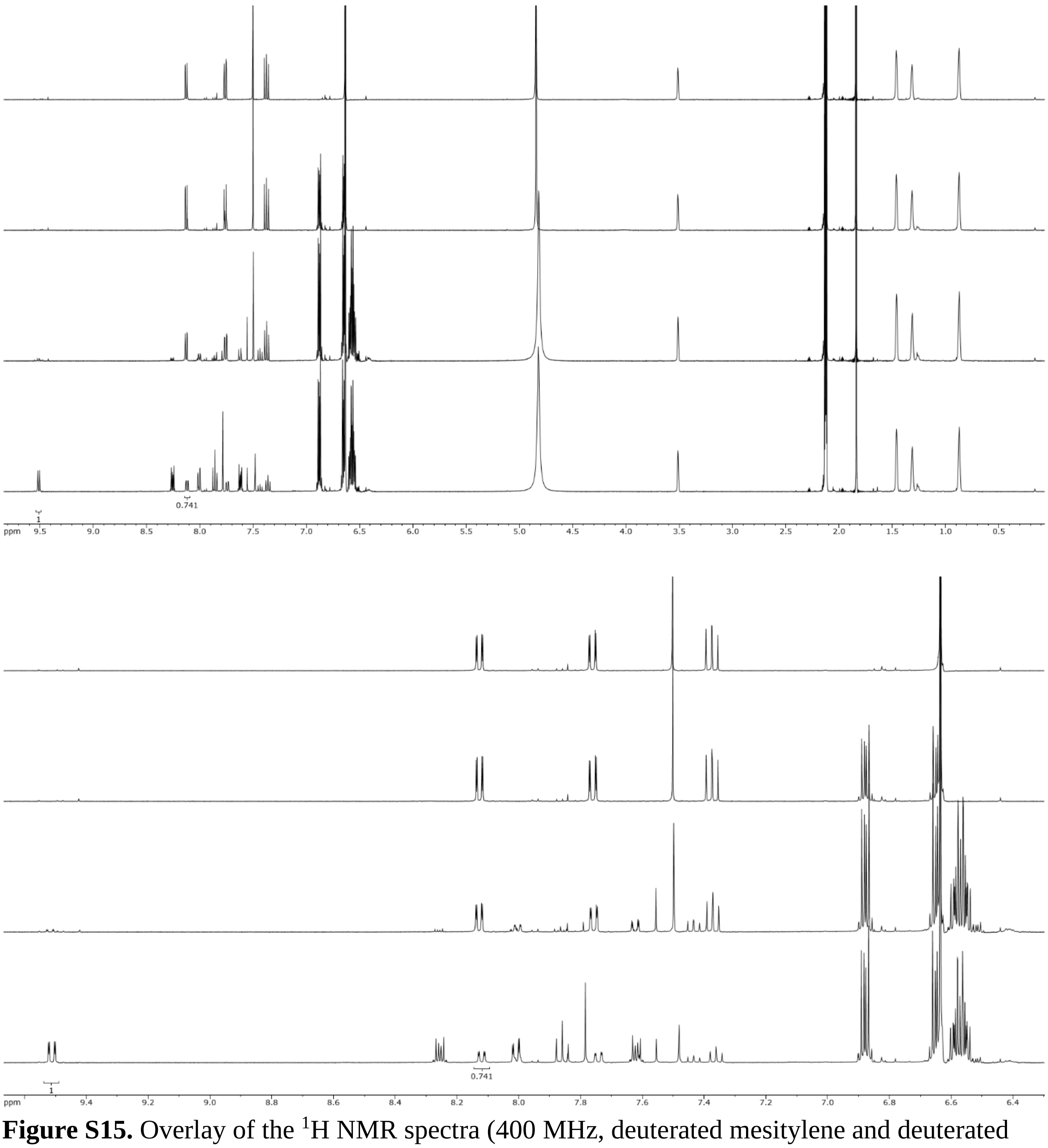

Figure S15. Overlay of the ^{1}H NMR spectra (400 MHz, deuterated mesitylene and deuterated *n*-butanol 1:1 (*v*/*v*), room temperature) of pyrene-4,5-dione (top), upon addition of catechol (2.2 equiv.; 2nd from top), upon addition of *o*-phenylenediamine (2.2 equiv.; 2nd from bottom), and after 1 h of *o*-phenylenediamine addition (bottom), respectively.

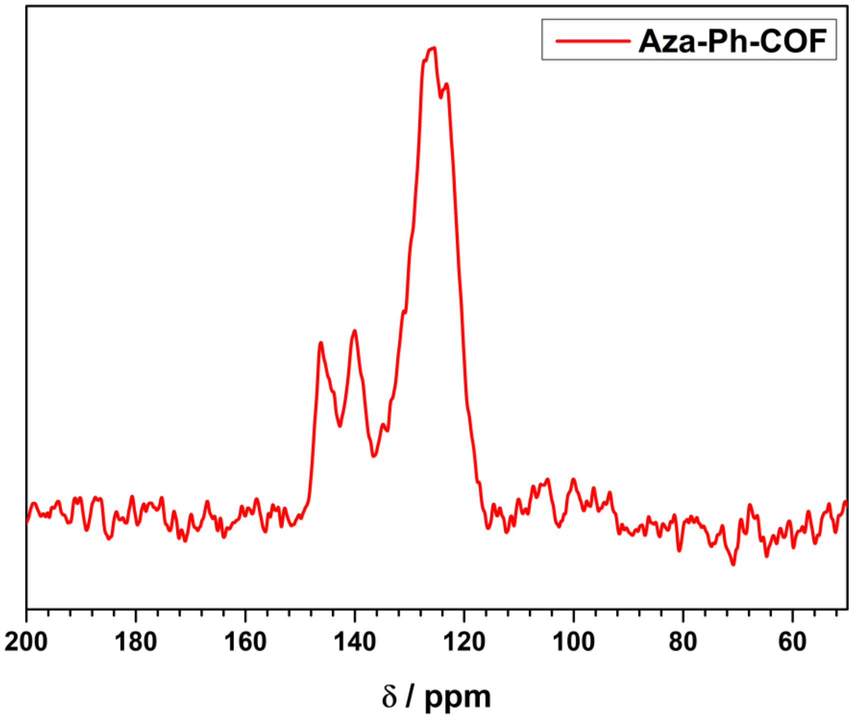

Figure S16. Solid-state ^{1}H-^{13}C CP-MAS NMR spectrum of Aza-Ph-COF. The absence of a carbonyl resonance at ~176 ppm, as seen in Dione-COF[3], provides additional evidence supporting the full conversion of the dione moieties in Aza-Ph-COF.

4. Fourier-transform (FT-IR) spectra

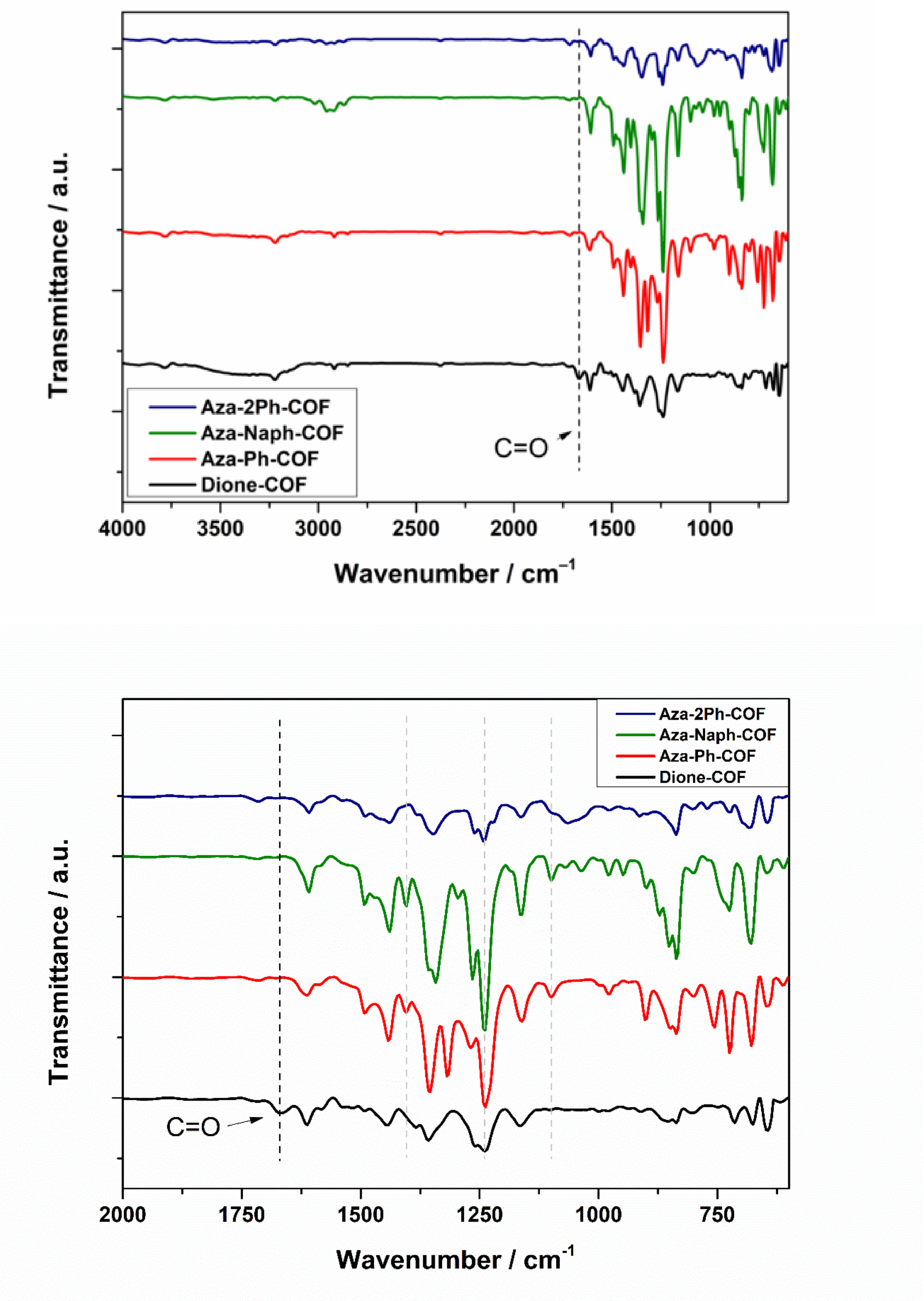

Figure S17. FT-IR spectrum of Dione-COF (black), Aza-Ph-COF (red), Aza-Naph-COF (green), and Aza-2Ph-COF (blue) (top); and a zoom-in (bottom), with the bands indicated for the C=O bond (black dashed line) and the pyrazaacene moieties (gray dashed lines at 1404, 1238, and 1099 cm^{-1}).

5. Structural analysis

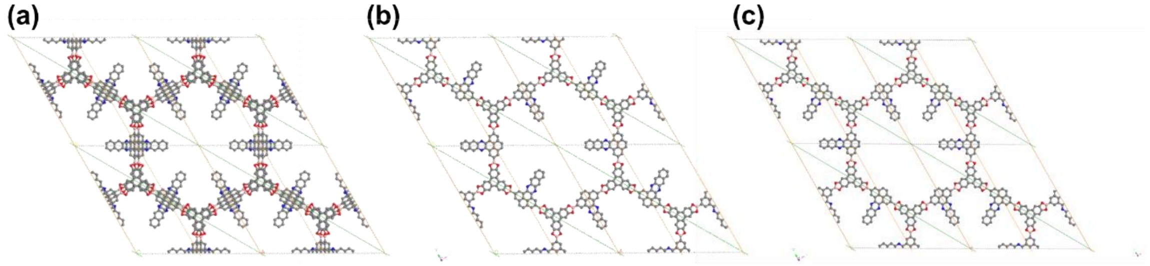

Figure S18. (**a**) A 2x2 Aza-Naph-COF unit cell display along the c-axis as a representative for an AA_{AP}, (**b**) high symmetry AA_{P} with alternating moieties in a pore (**c**) and low symmetry, *P*1, AA_{P} where three adjacent moieties in a pore with the Miller planes (110) and (200) highlighted by green and orange lines, respectively.

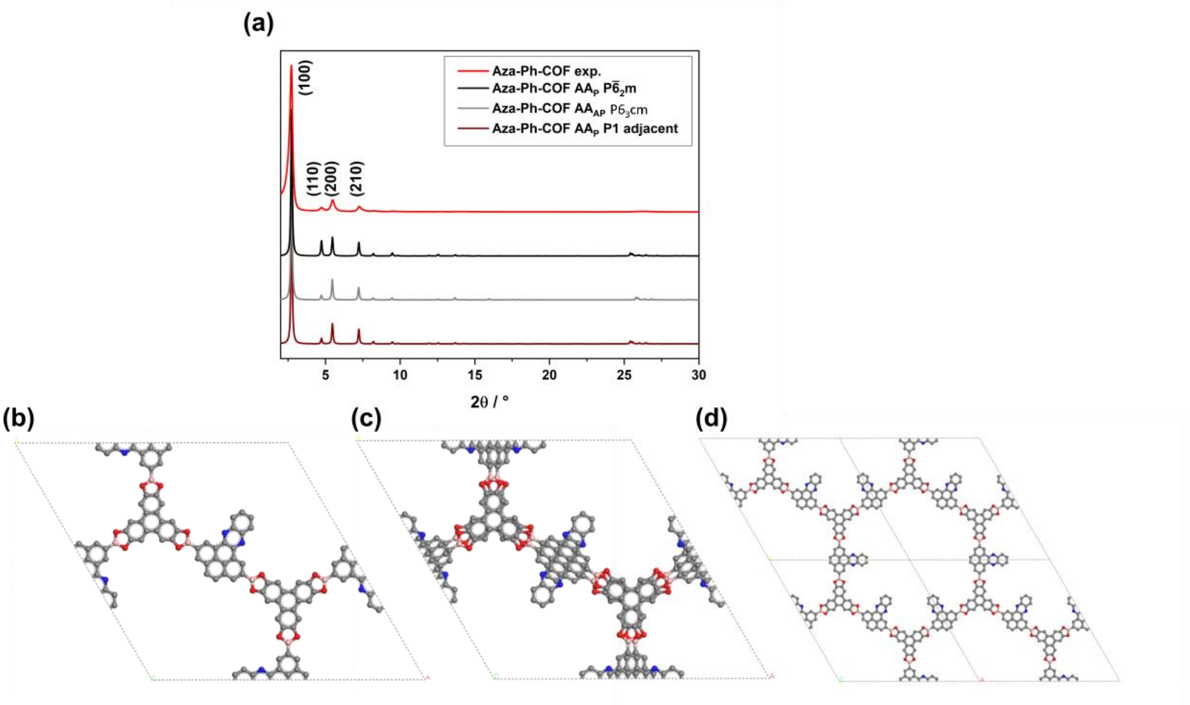

Figure S19. (**a**) Experimentally obtained and the simulated PXRD patterns for an AA_{P} (space group 189) and AA_{AP} (space group 186) layer arrangement of Aza-Ph-COF; (**b**) and (**c**) view of one unit cell for a high-symmetry AA_{AP} and AA_{P} layer arrangement. (**d**) A 2x2 unit cell display for AA_{P} *P*1 symmetry model.

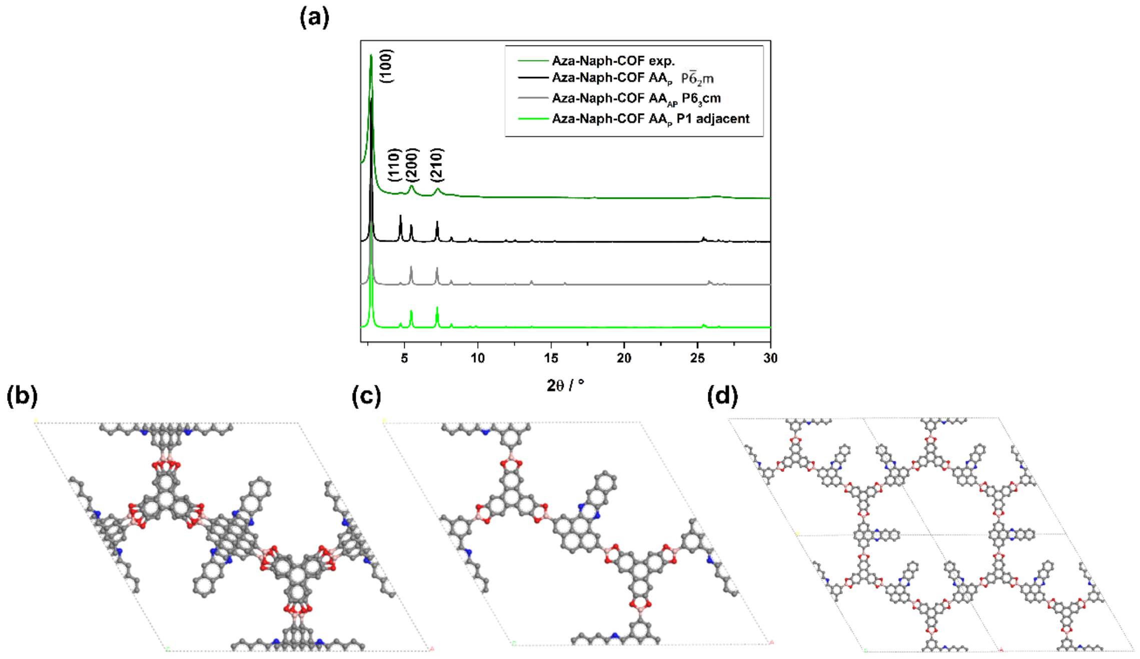

Figure S20. (**a**) Experimentally obtained and the simulated PXRD patterns for an AA$_P$ (space group 189) and AA$_{AP}$ (space group 186) layer arrangement of Aza-Naph-COF; (**b**) and (**c**) view of one unit cell for an AA$_P$ and AA$_{AP}$ layer arrangement, respectively. (**d**) A 2x2 unit cell display for AA$_P$ P1 symmetry model.

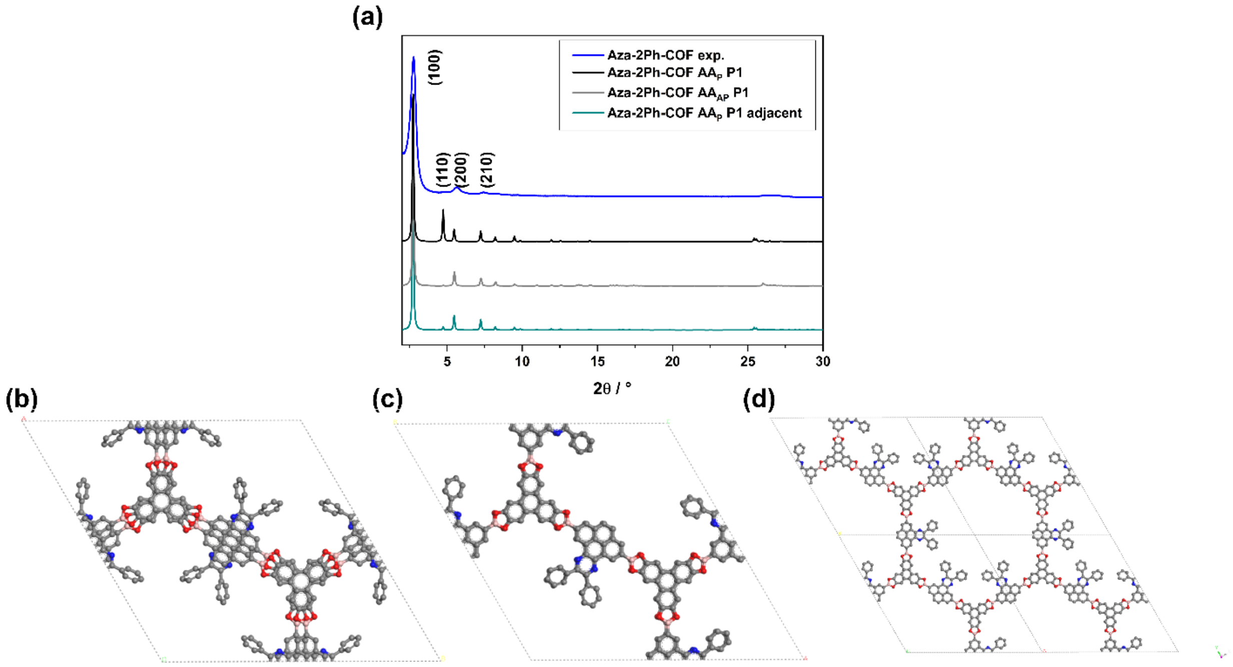

Figure S21. (**a**) Experimentally obtained and the simulated PXRD patterns for an AA$_P$ and AA$_{AP}$ layer arrangement of Aza-2Ph-COF; (**b**) and (**c**) view of one unit cell for an AA$_P$ and AA$_{AP}$ layer arrangement, respectively. (**d**) A 2x2 unit cell display for AA$_P$ *P*1 symmetry model.

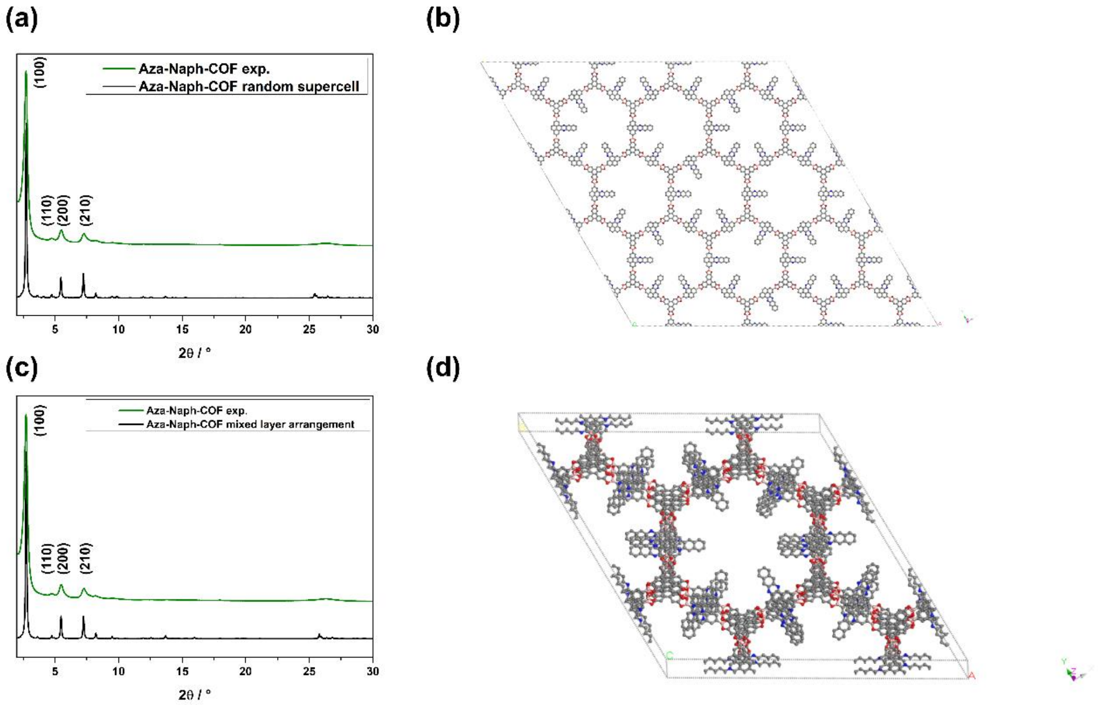

Figure S22. (**a**) Experimentally obtained and the simulated PXRD pattern for a supercell with random layer arrangement of Aza-Naph-COF. (**b**) View of one supercell for the random layer arrangement, respectively. (c) Experimentally obtained and the simulated PXRD pattern of a mixed $AA_{AP/P}$ layer arrangement of Aza-Naph-COF. (**d**) View of unit cell for the mixed $AA_{AP/P}$ layer arrangement.

	Aza-Ph-COF		Aza-2Ph-COF		Aza-Naph-COF	
Stacking	Parallel	AP	Parallel	AP	Parallel	AP
AA	0.0	−52.8	0.0	−1.5	0.0	25.2
Slip AA	−61.9	−53.6	1.6	5.9	−19.2	22.7

Table S1: Lattice energies (kcal/mol), relative to parallel azaacene moieties and AA stacking of Aza-COFs obtained from DFTB geometry optimization. Parallel and antiparallel (AP) refer to the orientation of azaacene moieties across layers as discussed in the manuscript.

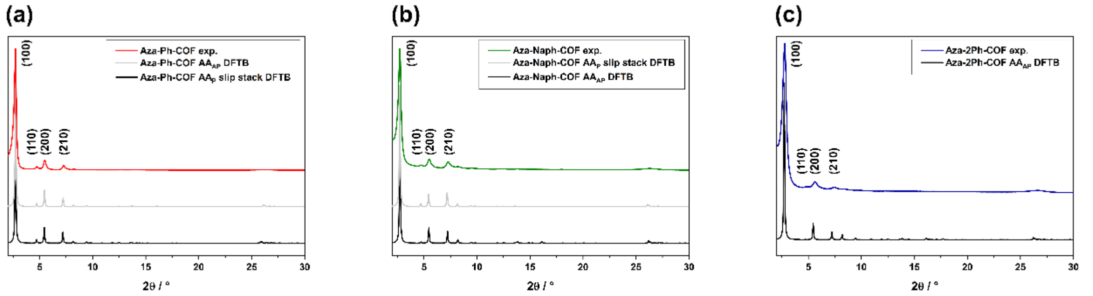

Figure S23. (**a**), (**b**), and (**c**) Experimentally obtained and the simulated PXRD patterns of DFTB calculations for an AA$_{AP}$ and AA$_P$ slip-stacked layer arrangement of Aza-Ph-COF, Aza-Naph- and Aza-2Ph-COF, respectively.

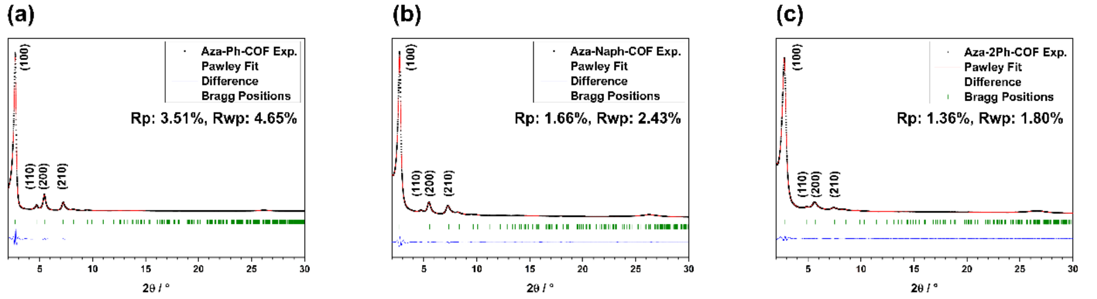

Figure S24. (**a**), (**b**), and (**c**) Experimental PXRD patterns (black dots) of Aza-Ph-, Aza-Naph-, and Aza-2Ph-COF using a parallel alignment of the layers for Aza-Ph-COF and antiparallel alignment of the layers for Aza-Naph- and Aza-2Ph-COF, respectively. Pawley refinement (red line), difference plot between the experimental data and the Pawley-refined PXRD pattern (blue line), Bragg positions are indicated by green ticks.

Unit cell parameters (*P*1) and atomic coordinates for Aza-Ph-COF (AAP slip-stack model)

a = b = 37.5 Å, c = 6.9 Å

α = 89.99° β = 89.96°, ɣ = 119.86°

Atom	a/x	b/y	c/z
C1	0.69639	0.46893	0.25849
C2	0.65677	0.43284	0.26044
C3	0.62086	0.43626	0.26457
C4	0.62521	0.47513	0.26585
C5	0.66403	0.5107	0.26347
C6	0.69975	0.50833	0.26013
O7	0.65859	0.54574	0.26353
B8	0.61609	0.52943	0.26518
O9	0.59442	0.48657	0.26762
C10	0.59432	0.55379	0.2609
C11	0.61494	0.59692	0.25463
C12	0.59376	0.61913	0.24645
C13	0.54991	0.59731	0.24435
C14	0.52839	0.5535	0.25134
C15	0.55085	0.53298	0.25957
C16	0.61482	0.66345	0.23969
C17	0.48322	0.53056	0.24952
C18	0.42299	0.47034	0.25515
N19	0.46424	0.48965	0.25716
C20	0.40001	0.42634	0.26383
C21	0.35778	0.40555	0.26217
C22	0.65287	0.39206	0.25767
C23	0.68884	0.3883	0.25326
C24	0.68515	0.34882	0.2506
C25	0.64617	0.31462	0.25269
C26	0.61073	0.31824	0.25691
C27	0.61336	0.35641	0.2593
O28	0.57547	0.27809	0.25838
B29	0.59146	0.25156	0.25514
O30	0.63432	0.27228	0.25148
C31	0.56677	0.20552	0.25541
C32	0.52362	0.18319	0.25704
C33	0.50135	0.13982	0.2564
C34	0.52313	0.11764	0.25419
C35	0.56691	0.13974	0.25302
C36	0.58749	0.18267	0.25362
C37	0.45704	0.11667	0.25769
C38	0.58985	0.11744	0.25038
C39	0.65008	0.11725	0.24583

N40	0.63077	0.13927	0.24957
C41	0.69409	0.13819	0.24466
C42	0.71489	0.11667	0.23977
C43	0.72953	0.42516	0.25218
C44	0.73334	0.46499	0.25473
C45	0.77291	0.50051	0.25423
C46	0.80708	0.49545	0.25187
C47	0.80331	0.45633	0.24953
C48	0.76509	0.42105	0.24932
O49	0.84329	0.46075	0.24826
B50	0.87003	0.50311	0.25013
O51	0.15050	0.52556	0.25207
C52	0.91603	0.52396	0.25045
C53	0.93841	0.50273	0.24991
C54	0.98142	0.5231	0.25037
C55	0.00386	0.56695	0.25137
C56	0.98204	0.58906	0.25201
C57	0.93882	0.56708	0.25158
C58	0.00391	0.50023	0.2499
C59	0.00498	0.63336	0.25308
C60	0.35582	0.79545	0.25145
C61	0.39539	0.7988	0.24575
C62	0.43124	0.8381	0.24428
C63	0.42676	0.87258	0.24802
C64	0.38789	0.86938	0.25329
C65	0.35229	0.83138	0.25534
O66	0.39301	0.90967	0.25541
B67	0.43547	0.93586	0.25118
O68	0.45735	0.9148	0.24682
C69	0.4569	0.98192	0.25141
C70	0.43613	1.00439	0.2537
C71	0.45718	1.04776	0.25439
C72	0.50103	1.06976	0.25294
C73	0.52263	1.04748	0.25019
C74	0.50033	1.00455	0.24941
C75	0.43608	1.07107	0.25665
C76	0.56775	1.0696	0.24814
C77	0.62794	1.06933	0.24238
N78	0.58669	1.04753	0.24405
C79	0.65081	1.0481	0.23712
C80	0.69303	1.06934	0.23561
C81	0.39898	0.7617	0.24093
C82	0.36284	0.72216	0.24222
C83	0.36624	0.6862	0.23749
C84	0.4051	0.69059	0.23167
C85	0.44069	0.72941	0.23037

C86	0.43835	0.76512	0.2348
O87	0.47576	0.72411	0.2252
B88	0.45953	0.68161	0.2243
O89	0.41667	0.65986	0.22736
C90	0.48407	0.66003	0.22403
C91	0.5272	0.68074	0.22455
C92	0.54951	0.65962	0.23018
C93	0.52778	0.61575	0.23573
C94	0.48399	0.59417	0.23357
C95	0.46338	0.61655	0.2275
C96	0.59382	0.68075	0.23188
C97	0.4611	0.54901	0.23977
C98	0.40085	0.48883	0.24463
N99	0.42019	0.53008	0.23713
C100	0.35684	0.46596	0.24281
C101	0.33596	0.42375	0.25123
C102	0.32224	0.71864	0.248
C103	0.31881	0.75485	0.25241
C104	0.27951	0.7515	0.25677
C105	0.24509	0.71264	0.25608
C106	0.24841	0.67702	0.25197
C107	0.2864	0.67928	0.24821
O108	0.2082	0.64198	0.2514
B109	0.18189	0.65821	0.25471
O110	0.20284	0.70109	0.2581
C111	0.13587	0.63359	0.2539
C112	0.11353	0.59011	0.25219
C113	0.07053	0.56763	0.2513
C114	0.04806	0.58905	0.25196
C115	0.06986	0.63291	0.2533
C116	0.11309	0.65404	0.25433
C117	0.04819	0.5224	0.25003
C118	0.04684	0.65427	0.25369
N119	0.98217	0.45929	0.24948
C120	0.0042	0.43996	0.24907
C121	0.04843	0.46213	0.24871
N122	0.07019	0.5034	0.24924
C123	0.06969	0.43927	0.24804
C124	0.04852	0.39702	0.24804
C125	0.00488	0.37512	0.24862
C126	0.98334	0.39592	0.24904
C169	0.68353	0.44736	0.75844
C170	0.64673	0.40836	0.75966
C171	0.60791	0.40619	0.76359
C172	0.60683	0.44257	0.76577
C173	0.64308	0.48086	0.76444

C174	0.68143	0.48403	0.76096
O175	0.63272	0.51247	0.76574
B176	0.58986	0.4917	0.76706
O177	0.57254	0.44907	0.76799
C178	0.56596	0.51393	0.76411
C179	0.58654	0.55708	0.75894
C180	0.56547	0.57932	0.75111
C181	0.52163	0.5574	0.74881
C182	0.5001	0.51359	0.75541
C183	0.52253	0.49299	0.76286
C184	0.58673	0.62362	0.74435
C185	0.45492	0.49076	0.75395
C186	0.39458	0.43065	0.76003
N187	0.43583	0.44984	0.76157
C188	0.37146	0.38664	0.76851
C189	0.32923	0.36595	0.76733
C190	0.64911	0.37059	0.75665
C191	0.68817	0.37279	0.75253
C192	0.69064	0.33629	0.74989
C193	0.65445	0.29884	0.75178
C194	0.61605	0.29671	0.7557
C195	0.61262	0.3318	0.75804
O196	0.58471	0.25476	0.7571
B197	0.60579	0.23287	0.75415
O198	0.64834	0.25823	0.75068
C199	0.58378	0.18681	0.75426
C200	0.54066	0.1645	0.75554
C201	0.51834	0.12125	0.75473
C202	0.54012	0.0991	0.75249
C203	0.58393	0.12115	0.75138
C204	0.60466	0.16415	0.7524
C205	0.47405	0.09855	0.75572
C206	0.60657	0.0985	0.7482
C207	0.66662	0.0979	0.74223
N208	0.64749	0.12011	0.74673
C209	0.71061	0.11863	0.73973
C210	0.73124	0.09695	0.73353
C211	0.72583	0.41282	0.75175
C212	0.72356	0.44965	0.75463
C213	0.76001	0.48849	0.75444
C214	0.79742	0.48955	0.75202
C215	0.79964	0.45331	0.7494
C216	0.76459	0.41493	0.74891
O217	0.84156	0.46374	0.74811
B218	0.86341	0.50659	0.75024
O219	0.83802	0.52389	0.75239

C220	0.90943	0.53056	0.75059
C221	0.93193	0.50953	0.75002
C222	0.97482	0.52991	0.75048
C223	0.99708	0.57373	0.75149
C224	0.97517	0.59578	0.75214
C225	0.93186	0.5737	0.75173
C226	0.6696	0.50673	0.74999
C227	0.99843	0.6401	0.75322
C228	0.35487	0.7799	0.75039
C229	0.39159	0.77754	0.74446
C230	0.43047	0.81394	0.74282
C231	0.43166	0.85143	0.7465
C232	0.39546	0.85371	0.75191
C233	0.35707	0.81872	0.75418
O234	0.40598	0.89571	0.75411
B235	0.44884	0.91748	0.74988
O236	0.46603	0.89201	0.74533
C237	0.47299	0.96353	0.75011
C238	0.45265	0.98641	0.75201
C239	0.47397	1.02968	0.75262
C240	0.51781	1.05124	0.75123
C241	0.53907	1.0286	0.74863
C242	0.51642	0.98564	0.74825
C243	0.45292	1.053	0.75463
C244	0.58423	1.0506	0.74605
C245	0.64426	1.04999	0.73886
N246	0.603	1.02839	0.74139
C247	0.66694	1.02857	0.73246
C248	0.70917	1.04962	0.72952
C249	0.38913	0.73742	0.73983
C250	0.35014	0.70057	0.7419
C251	0.34811	0.66179	0.73788
C252	0.38456	0.66083	0.73194
C253	0.42279	0.69715	0.72951
C254	0.42582	0.73544	0.73329
O255	0.4545	0.687	0.72445
B256	0.43386	0.64414	0.72491
O257	0.39124	0.62667	0.72855
C258	0.45622	0.62036	0.72588
C259	0.49936	0.64092	0.72661
C260	0.52154	0.61978	0.73348
C261	0.49959	0.57593	0.73982
C262	0.4558	0.55448	0.7377
C263	0.43524	0.57694	0.73067
C264	0.56583	0.64097	0.73564
C265	0.43288	0.50932	0.74454

C266	0.37254	0.44923	0.74998
N267	0.39197	0.49048	0.74236
C268	0.32852	0.42648	0.74867
C269	0.30751	0.38426	0.75707
C270	0.31242	0.70305	0.74781
C271	0.31487	0.74226	0.75178
C272	0.27859	0.74505	0.75618
C273	0.24104	0.70903	0.75591
C274	0.23856	0.67046	0.75227
C275	0.27348	0.66671	0.74854
O276	0.1965	0.63942	0.75192
B277	0.17494	0.66082	0.75485
O278	0.20062	0.70335	0.75785
C279	0.12893	0.63937	0.75402
C280	0.10618	0.59595	0.75226
C281	0.06329	0.57385	0.75137
C282	0.04124	0.59568	0.75206
C283	0.06339	0.63955	0.7534
C284	0.10671	0.66039	0.75445
C285	0.04106	0.5287	0.75011
C286	0.04033	0.6609	0.7538
N287	0.97495	0.46579	0.74958
C288	0.99686	0.44636	0.74917
C289	0.04111	0.4684	0.74881
N290	0.06302	0.50966	0.74933
C291	0.06226	0.44543	0.74814
C292	0.04096	0.40319	0.74815
C293	0.99731	0.38141	0.74874
C294	0.97588	0.40231	0.74915

Unit cell parameters (*P*1) and atomic coordinates for Aza-Naph-COF (AA$_{AP}$ model)

a = b = 37.4 Å, c = 6.8 Å

α = 90.000° β = 89.987°, ɣ = 120.000°

Atom	a/x	b/y	c/z
C1	0.67243	0.37552	0.49657
C2	0.62544	0.29882	0.49652
C3	0.70212	0.32852	0.49635
C4	0.32766	0.62615	0.99526
C5	0.37469	0.70285	0.99683
C6	0.298	0.67317	0.99646
C7	0.67245	0.29869	0.99674
C8	0.62544	0.32838	0.99652
C9	0.70213	0.37538	0.99661
C10	0.32771	0.70304	0.49699
C11	0.37468	0.67331	0.49545
C12	0.29798	0.62635	0.49565
C13	0.59712	0.34656	0.49584
C14	0.65434	0.25244	0.49743
C15	0.74853	0.4038	0.49676
C16	0.40297	0.65507	0.99371
C17	0.34578	0.74923	0.99871
C18	0.25155	0.5979	0.99588
C19	0.59714	0.25233	0.99742
C20	0.65434	0.40367	0.9964
C21	0.74856	0.34652	0.9961
C22	0.40301	0.7494	0.49863
C23	0.34573	0.59801	0.49362
C24	0.25162	0.65529	0.49614
C25	0.60404	0.38654	0.49604
C26	0.61435	0.21941	0.49785
C27	0.78156	0.39683	0.49649
C28	0.39602	0.61508	0.99282
C29	0.38577	0.78226	0.99947
C30	0.21854	0.6049	0.99603
C31	0.60407	0.21927	0.99778
C32	0.61435	0.39672	0.99634
C33	0.78158	0.38654	0.99624
C34	0.39607	0.78245	0.49954
C35	0.38571	0.60493	0.4928
C36	0.21857	0.6153	0.49603
O37	0.64118	0.45757	0.49679
O38	0.54332	0.18559	0.4981
O39	0.81536	0.35958	0.49582
O40	0.35885	0.54405	0.99286

O41	0.4568	0.8161	0.9993
O42	0.18479	0.6422	0.99604
O43	0.64123	0.1854	0.99754
O44	0.54331	0.35952	0.99639
O45	0.81536	0.45757	0.9968
O46	0.35888	0.81629	0.49971
O47	0.45674	0.64208	0.49244
O48	0.18471	0.54427	0.49618
B49	0.59886	0.44334	0.4964
B50	0.55752	0.15749	0.49859
B51	0.8435	0.40188	0.49633
B52	0.40117	0.55825	0.99218
B53	0.44258	0.84419	0.99954
B54	0.15662	0.59991	0.99602
B55	0.59891	0.15733	0.99769
B56	0.5575	0.40183	0.99641
B57	0.84351	0.44343	0.99639
B58	0.40119	0.84438	0.50028
B59	0.44253	0.59977	0.49209
B60	0.1566	0.55848	0.49637
C61	0.57846	0.46912	0.49639
C62	0.53174	0.11132	0.49884
C63	0.88967	0.42222	0.49658
C64	0.42155	0.53246	0.99248
C65	0.46834	0.89036	0.99839
C66	0.11045	0.57961	0.99596
C67	0.57849	0.11115	0.997
C68	0.5317	0.42221	0.99621
C69	0.88968	0.46927	0.99623
C70	0.42157	0.89055	0.50003
C71	0.4683	0.57936	0.49269
C72	0.11043	0.53267	0.49672
C73	0.59941	0.51244	0.49709
C74	0.48843	0.089	0.50077
C75	0.91204	0.40127	0.49743
C76	0.4006	0.48914	0.99246
C77	0.51165	0.9127	0.99537
C78	0.08811	0.60059	0.99505
C79	0.59945	0.0888	0.99427
C80	0.48838	0.40125	0.99614
C81	0.91205	0.51259	0.99537
C82	0.40061	0.91289	0.50159
C83	0.51162	0.60031	0.49386
C84	0.08807	0.48935	0.49771
C85	0.57827	0.5349	0.49663
C86	0.46599	0.04541	0.50069

C87	0.95565	0.42242	0.49769
C88	0.42173	0.46667	0.99338
C89	0.53407	0.95628	0.99404
C90	0.0445	0.57947	0.99496
C91	0.57829	0.04521	0.99354
C92	0.46594	0.42241	0.99525
C93	0.95566	0.53506	0.99511
C94	0.42176	0.95648	0.50103
C95	0.53406	0.57915	0.49507
C96	0.04447	0.46687	0.49785
C97	0.53422	0.51306	0.49565
C98	0.48784	0.02322	0.49858
C99	0.97786	0.46647	0.49696
C100	0.46578	0.48851	0.99414
C101	0.51221	0.97847	0.996
C102	0.02226	0.53541	0.99583
C103	0.53424	0.02303	0.99576
C104	0.48778	0.46646	0.9949
C105	0.97786	0.51321	0.99587
C106	0.46581	0.97867	0.49877
C107	0.51221	0.5351	0.49493
C108	0.02225	0.4887	0.497
C109	0.51244	0.46907	0.49523
C110	0.53185	0.04541	0.49654
C111	0.95563	0.48825	0.49613
C112	0.48756	0.5325	0.99425
C113	0.46821	0.95627	0.99853
C114	0.04446	0.51361	0.9967
C115	0.51245	0.04524	0.99815
C116	0.53178	0.48825	0.99535
C117	0.95564	0.46921	0.99672
C118	0.4876	0.95646	0.49692
C119	0.46821	0.51332	0.49397
C120	0.04445	0.5327	0.49614
C121	0.5348	0.44837	0.49552
C122	0.55251	0.08844	0.49684
C123	0.91259	0.46588	0.49602
C124	0.46521	0.5532	0.99343
C125	0.44756	0.91324	0.99962
C126	0.0875	0.53596	0.99674
C127	0.53484	0.08828	0.99866
C128	0.55245	0.46586	0.99581
C129	0.9126	0.44853	0.99683
C130	0.46522	0.91342	0.49773
C131	0.44755	0.5357	0.49284
C132	0.0875	0.55341	0.49602

C133	0.59945	0.57942	0.49719
C134	0.42147	0.0221	0.50272
C135	0.97901	0.40126	0.49867
C136	0.59945	0.42215	0.99379
C137	0.57859	0.9796	0.99083
C138	0.02115	0.60064	0.99398
C139	0.59947	0.02189	0.9906
C140	0.42141	0.40124	0.99476
C141	0.97903	0.57958	0.99407
C142	0.40058	0.97981	0.50284
C143	0.57858	0.60033	0.49646
C144	0.02114	0.42235	0.49878
C145	0.46711	0.44587	0.49426
C146	0.55508	0.02328	0.49445
C147	0.97773	0.53358	0.49538
C148	0.5329	0.5557	0.99529
C149	0.44498	0.97839	0.99998
C150	0.02234	0.46828	0.99746
C151	0.46711	0.02314	0.99994
C152	0.55501	0.53359	0.99537
C153	0.97776	0.44599	0.99743
C154	0.53293	0.97854	0.49457
C155	0.44499	0.46797	0.49396
C156	0.02231	0.5559	0.49537
C157	0.40665	0.38529	0.49218
C158	0.61561	0.02328	0.49217
C159	0.97759	0.59403	0.49422
C160	0.59337	0.61627	0.99677
C161	0.38443	0.97837	0.00193
C162	0.02242	0.4078	0.99851
C163	0.40666	0.02329	0.00224
C164	0.61557	0.59408	0.99586
C165	0.59325	0.38544	0.99847
C166	0.59336	0.97837	0.49209
C167	0.38443	0.40747	0.49274
C168	0.02234	0.61643	0.49422
N169	0.44821	0.40488	0.49339
N170	0.59608	0.04531	0.49291
N171	0.95563	0.55247	0.49475
N172	0.55181	0.59669	0.99618
N173	0.40398	0.95636	0.00109
N174	0.04441	0.44937	0.99805
N175	0.44822	0.04524	0.00135
N176	0.59601	0.55252	0.99542
N177	0.95569	0.405	0.99798
N178	0.5518	0.95643	0.49294

N179	0.40399	0.44903	0.49357
N180	0.04435	0.59689	0.49474
C181	0.3841	0.34167	0.48981
C182	0.65924	0.04423	0.49249
C183	0.95655	0.61662	0.4938
C184	0.61593	0.65989	0.99825
C185	0.34079	0.95741	0.00206
C186	0.04344	0.38519	0.9989
C187	0.38411	0.04437	0.00298
C188	0.65921	0.61673	0.99551
C189	0.95665	0.34181	0.99883
C190	0.6159	0.95727	0.49215
C191	0.3408	0.3848	0.49181
C192	0.04334	0.66005	0.49382
C193	0.34082	0.31938	0.4883
C194	0.68142	0.02311	0.49366
C195	0.97758	0.65991	0.4935
C196	0.65921	0.68217	0.99855
C197	0.31861	0.97851	0.0022
C198	0.02238	0.3419	0.99918
C199	0.34083	0.02338	0.00279
C200	0.68141	0.66002	0.99679
C201	0.97768	0.31956	0.99916
C202	0.65918	0.97824	0.49343
C203	0.31861	0.34151	0.48969
C204	0.02228	0.68228	0.49351
C205	0.63168	0.34067	0.49622
C206	0.66027	0.2929	0.49672
C207	0.70804	0.36927	0.4966
C208	0.36842	0.66099	0.99519
C209	0.33985	0.70877	0.99731
C210	0.29205	0.6324	0.99596
C211	0.63169	0.29278	0.99686
C212	0.66027	0.36913	0.99652
C213	0.70806	0.34055	0.99641
C214	0.36846	0.70894	0.4971
C215	0.33983	0.63257	0.49494
C216	0.29209	0.6612	0.49614
C217	0.64405	0.42093	0.49655
C218	0.57998	0.22507	0.49756
C219	0.77588	0.3568	0.49598
C220	0.35601	0.58071	0.99337
C221	0.42013	0.77661	0.99905
C222	0.22425	0.64494	0.99611
C223	0.64408	0.22491	0.99754
C224	0.57999	0.3567	0.99634

C225	0.77589	0.42088	0.99668
C226	0.35606	0.77681	0.49908
C227	0.42009	0.64494	0.49308
C228	0.2242	0.58092	0.49595
C229	0.67846	0.41611	0.49675
C230	0.58483	0.2643	0.49686
C231	0.73663	0.32242	0.4959
C232	0.32161	0.58555	0.99446
C233	0.41529	0.73738	0.99789
C234	0.26351	0.6793	0.99637
C235	0.67849	0.26413	0.9971
C236	0.58483	0.32231	0.99639
C237	0.73663	0.41599	0.99684
C238	0.32166	0.73758	0.49793
C239	0.41528	0.67935	0.49433
C240	0.26344	0.58576	0.49564
O241	0.57499	0.40035	0.4959
O242	0.60051	0.17656	0.4985
O243	0.82444	0.42582	0.49667
O244	0.42505	0.60125	0.99188
O245	0.39959	0.82511	0.00009
O246	0.17565	0.57594	0.99603
O247	0.57502	0.17643	0.99808
O248	0.6005	0.42573	0.99636
O249	0.82446	0.40043	0.99607
O250	0.4251	0.8253	0.50044
O251	0.39953	0.57589	0.49198
O252	0.1757	0.60147	0.49622
C253	0.31768	0.27506	0.48543
C254	0.72574	0.04412	0.49567
C255	0.95648	0.68313	0.49325
C256	0.68237	0.72649	0.00059
C257	0.27427	0.95749	0.0015
C258	0.04345	0.31865	0.99946
C259	0.31768	0.04456	0.00279
C260	0.72574	0.68332	0.99675
C261	0.68229	0.27524	0.99945
C262	0.68231	0.95704	0.49513
C263	0.27429	0.3182	0.48862
C264	0.04338	0.7266	0.49325
C265	0.27536	0.2541	0.48436
C266	0.74654	0.02258	0.49748
C267	0.97792	0.72546	0.493
C268	0.72469	0.74744	0.00085
C269	0.25346	0.97901	0.00129
C270	0.02198	0.27632	0.99973

C271	0.27535	0.02318	0.00198
C272	0.74656	0.72565	0.99879
C273	0.97797	0.25433	0.99974
C274	0.72464	0.97841	0.49719
C275	0.25348	0.27588	0.48611
C276	0.02193	0.74748	0.493

Unit cell parameters (*P*1) and atomic coordinates for Aza-2Ph-COF (AA_{AP} model)

a = b = 37.3 Å, c = 6.8 Å

α = β = 90.000°, ɣ = 120.000°

Atom	a/x	b/y	c/z
C1	0.41626	0.39754	0.24773
C2	0.46085	0.4187	0.24573
C3	0.48282	0.46284	0.24816
C4	0.54984	0.52994	0.24918
C5	0.5269	0.48468	0.2469
C6	0.54758	0.46224	0.24475
C7	0.52667	0.41859	0.244
C8	0.48324	0.39752	0.244
N9	0.59125	0.54943	0.25423
C10	0.61082	0.59064	0.25623
C11	0.65692	0.61146	0.2758
B12	0.55241	0.39811	0.24519
O13	0.59545	0.42202	0.24458
C14	0.60921	0.39293	0.24798
C15	0.57477	0.35289	0.25037
O16	0.53809	0.35576	0.24839
C17	0.5795	0.31839	0.25398
C18	0.62015	0.32442	0.25519
C19	0.65505	0.36522	0.25266
C20	0.64924	0.39987	0.24897
C21	0.59539	0.01821	0.25373
C22	0.57416	0.04155	0.25591
C23	0.53002	0.01926	0.25199
C24	0.46281	0.01895	0.25089
C25	0.50807	0.04136	0.25402
C26	0.53043	0.08447	0.25882
C27	0.57408	0.10731	0.26231
C28	0.59527	0.08509	0.26163
N29	0.44326	0.04079	0.25905
C30	0.40206	0.019	0.25627
C31	0.38049	0.04357	0.27901
B32	0.59435	0.1535	0.26381
O33	0.57022	0.17239	0.26271
C34	0.59912	0.21531	0.26179
C35	0.63926	0.22112	0.26265
O36	0.63667	0.18172	0.26417
C37	0.67361	0.26041	0.26191
C38	0.66729	0.29482	0.25938
C39	0.62644	0.2888	0.25844
C40	0.59192	0.24823	0.26012

C41	0.97365	0.57587	0.24956
C42	0.95034	0.53128	0.2496
C43	0.97265	0.50944	0.25117
C44	0.97305	0.44256	0.25276
C45	0.95059	0.46537	0.25162
C46	0.90745	0.44455	0.25115
C47	0.88456	0.46532	0.25033
C48	0.90676	0.50873	0.2494
N49	0.95132	0.40116	0.25821
C50	0.97317	0.3818	0.25793
C51	0.94836	0.33564	0.27628
B52	0.83834	0.43945	0.25106
O53	0.81931	0.39645	0.25448
C54	0.77642	0.3826	0.25475
C55	0.77072	0.41698	0.25136
O56	0.81022	0.45368	0.24883
C57	0.73147	0.41218	0.25077
C58	0.69695	0.37151	0.25363
C59	0.70291	0.33668	0.25711
C60	0.74341	0.34256	0.25769
C61	0.57391	0.97609	0.24775
C62	0.52936	0.9527	0.24361
C63	0.50745	0.97493	0.24562
C64	0.4405	0.97515	0.2435
C65	0.46339	0.95278	0.24142
C66	0.44269	0.90968	0.23564
C67	0.46356	0.8869	0.23436
C68	0.50696	0.90915	0.23787
N69	0.39911	0.95329	0.23497
C70	0.37966	0.97504	0.23745
C71	0.33349	0.95037	0.21624
B72	0.43766	0.84071	0.23265
O73	0.39463	0.8218	0.23151
C74	0.38064	0.7789	0.23394
C75	0.41501	0.77314	0.23595
O76	0.4518	0.81254	0.23486
C77	0.41011	0.73388	0.23879
C78	0.36938	0.69947	0.24035
C79	0.33453	0.70543	0.23833
C80	0.34053	0.74596	0.23471
C81	0.3948	0.41866	0.25439
C82	0.41612	0.46324	0.25576
C83	0.46028	0.48508	0.25254
C84	0.52758	0.55194	0.25038
C85	0.4823	0.52914	0.25326
C86	0.46001	0.54998	0.2557

C87	0.41636	0.5292	0.2576
C88	0.39512	0.48579	0.25813
N89	0.54719	0.59333	0.24533
C90	0.58842	0.61279	0.24398
C91	0.60951	0.65893	0.22561
B92	0.39601	0.55506	0.25679
O93	0.41998	0.59807	0.25334
C94	0.3909	0.61186	0.25117
C95	0.35083	0.57744	0.25386
O96	0.35366	0.54078	0.25773
C97	0.31632	0.58215	0.25216
C98	0.3224	0.62277	0.24741
C99	0.36317	0.65764	0.24467
C100	0.39786	0.65187	0.24657
C101	0.01582	0.597	0.25212
C102	0.03924	0.57583	0.25212
C103	0.01702	0.53169	0.25215
C104	0.0169	0.46458	0.25161
C105	0.0392	0.50982	0.25241
C106	0.08233	0.5322	0.25193
C107	0.1051	0.57583	0.25035
C108	0.08282	0.59696	0.25057
N109	0.03888	0.44519	0.24521
C110	0.01728	0.40398	0.24396
C111	0.0425	0.38313	0.22285
B112	0.15131	0.5962	0.24801
O113	0.17037	0.57225	0.25024
C114	0.21323	0.60131	0.24707
C115	0.21885	0.64135	0.24272
O116	0.17935	0.63854	0.24334
C117	0.25806	0.67584	0.23932
C118	0.29265	0.66978	0.2407
C119	0.28676	0.629	0.24541
C120	0.2463	0.59436	0.24858
C121	0.95949	0.31347	0.405
C122	0.93521	0.27066	0.42265
C123	0.89921	0.24897	0.31189
C124	0.88755	0.27049	0.18439
C125	0.91171	0.31335	0.16791
C126	0.03134	0.34971	0.09483
C127	0.05616	0.33183	0.07415
C128	0.09274	0.3469	0.18103
C129	0.10448	0.38027	0.3074
C130	0.07979	0.39833	0.32689
C131	0.39317	0.07954	0.16756
C132	0.37416	0.10331	0.18945

C133	0.3424	0.09193	0.32528
C134	0.32981	0.05661	0.43906
C135	0.3486	0.03267	0.41594
C136	0.3105	0.91486	0.33104
C137	0.26765	0.89106	0.31242
C138	0.24689	0.9019	0.17629
C139	0.26932	0.93671	0.05891
C140	0.31213	0.96073	0.07894
C141	0.59441	0.68084	0.3317
C142	0.61271	0.72365	0.31523
C143	0.64628	0.74554	0.19023
C144	0.66126	0.72422	0.08136
C145	0.64313	0.68144	0.09888
C146	0.67867	0.59591	0.1717
C147	0.72143	0.61375	0.18979
C148	0.74342	0.64735	0.31453
C149	0.72225	0.66284	0.42113
C150	0.67953	0.64514	0.40202
C151	0.46879	0.44361	0.74704
C152	0.51405	0.46646	0.74672
C153	0.53598	0.51051	0.74852
C154	0.60138	0.57698	0.74963
C155	0.58014	0.5324	0.74844
C156	0.6012	0.5099	0.74632
C157	0.58001	0.46648	0.74512
C158	0.53636	0.44565	0.74521
B159	0.60041	0.44068	0.74573
O160	0.64278	0.45498	0.74514
C161	0.64562	0.41831	0.74793
C162	0.60555	0.38388	0.74991
O163	0.57646	0.39767	0.74838
C164	0.59858	0.34387	0.75314
C165	0.63324	0.33805	0.75445
C166	0.67402	0.37294	0.75244
C167	0.68011	0.41358	0.74903
C168	0.55649	0.02045	0.75609
C169	0.53352	0.04279	0.75696
C170	0.48944	0.02066	0.75198
C171	0.42288	0.01948	0.75159
C172	0.46748	0.04286	0.75438
C173	0.48991	0.08641	0.75881
C174	0.53333	0.10868	0.76136
C175	0.55422	0.08591	0.76113
B176	0.55911	0.15488	0.76273
O177	0.54483	0.18295	0.76129
C178	0.58154	0.22245	0.76126

C179	0.61598	0.21681	0.76289
O180	0.60214	0.17394	0.76405
C181	0.65604	0.24983	0.76205
C182	0.66191	0.29031	0.75944
C183	0.62701	0.2962	0.7578
C184	0.58633	0.26168	0.75878
C185	0.97942	0.53094	0.75073
C186	0.95717	0.48571	0.75202
C187	0.97938	0.46386	0.75237
C188	0.98064	0.39858	0.75303
C189	0.95719	0.41972	0.75314
C190	0.91361	0.39857	0.75299
C191	0.89131	0.41968	0.75278
C192	0.91404	0.4633	0.75229
B193	0.8451	0.39934	0.75349
O194	0.81701	0.35702	0.75728
C195	0.77754	0.35428	0.75688
C196	0.78321	0.39434	0.75317
O197	0.82609	0.42335	0.75101
C198	0.75018	0.40136	0.75191
C199	0.70969	0.36674	0.75402
C200	0.70378	0.32596	0.75767
C201	0.73831	0.31983	0.75931
C202	0.53423	0.97668	0.74344
C203	0.48891	0.95427	0.74056
C204	0.4669	0.97634	0.74495
C205	0.40142	0.97735	0.7454
C206	0.42273	0.95407	0.7419
C207	0.40168	0.91052	0.73689
C208	0.42288	0.88832	0.73416
C209	0.46654	0.91116	0.73532
B210	0.40249	0.84212	0.73262
O211	0.36013	0.81403	0.73264
C212	0.35735	0.77454	0.73405
C213	0.39744	0.78019	0.73445
O214	0.42647	0.82308	0.73313
C215	0.40447	0.74718	0.73734
C216	0.36986	0.70668	0.7397
C217	0.32906	0.70078	0.73898
C218	0.3229	0.7353	0.73621
C219	0.4464	0.46544	0.75295
C220	0.47245	0.50931	0.75171
C221	0.51334	0.53264	0.7503
C222	0.57983	0.59803	0.74862
C223	0.53523	0.57678	0.75004
C224	0.51275	0.59785	0.75216

C225	0.46932	0.57669	0.75401
C226	0.44847	0.53306	0.75446
B227	0.44362	0.59721	0.75382
O228	0.45802	0.63957	0.74956
C229	0.42139	0.64251	0.74867
C230	0.38691	0.60249	0.75255
O231	0.40059	0.57335	0.75604
C232	0.3469	0.59559	0.75189
C233	0.34116	0.63027	0.74743
C234	0.37608	0.67103	0.74374
C235	0.41672	0.67703	0.74419
C236	0.02325	0.55299	0.75224
C237	0.04575	0.53022	0.75138
C238	0.02374	0.48615	0.75139
C239	0.02281	0.41974	0.75046
C240	0.04607	0.46433	0.75044
C241	0.08964	0.4869	0.75033
C242	0.11178	0.5303	0.75022
C243	0.08889	0.55106	0.7506
B244	0.15798	0.55618	0.74902
O245	0.18608	0.54193	0.75158
C246	0.22556	0.57863	0.74883
C247	0.21989	0.61302	0.74461
O248	0.177	0.59918	0.74481
C249	0.2529	0.65306	0.74093
C250	0.2934	0.65894	0.74202
C251	0.29931	0.62407	0.74644
C252	0.2648	0.58341	0.7498
N253	0.04494	0.59438	0.75791
C254	0.02305	0.6137	0.75771
C255	0.97895	0.5915	0.74341
N256	0.95739	0.55029	0.74423
C257	0.9537	0.61236	0.72337
C258	0.04786	0.65986	0.77603
C259	0.96468	0.64577	0.59552
C260	0.93993	0.66378	0.57695
C261	0.90362	0.64886	0.68579
C262	0.89201	0.61545	0.81175
C263	0.91663	0.59726	0.82912
C264	0.08438	0.68207	0.6667
C265	0.10852	0.72493	0.68216
C266	0.09699	0.74656	0.80968
C267	0.06119	0.72493	0.92183
C268	0.0369	0.6821	0.90513
N269	0.40546	0.44578	0.7625
C270	0.386	0.40455	0.76091

C271	0.40807	0.38263	0.73973
N272	0.44929	0.40225	0.73767
C273	0.38739	0.3367	0.71376
C274	0.34003	0.38354	0.78556
C275	0.35305	0.31454	0.59051
C276	0.33563	0.27202	0.56466
C277	0.35211	0.25059	0.66132
C278	0.38647	0.27217	0.78233
C279	0.40404	0.31473	0.80709
C280	0.31778	0.39955	0.6894
C281	0.2751	0.38149	0.71203
C282	0.25369	0.34723	0.83378
C283	0.27535	0.33126	0.93255
C284	0.318	0.34919	0.90889
N285	0.55381	0.95484	0.73319
C286	0.59494	0.97674	0.74089
C287	0.61675	1.02051	0.76277
N288	0.59745	1.04234	0.76849
C289	0.66328	1.04394	0.78142
C290	0.61828	0.95353	0.72328
C291	0.6192	0.93552	0.54479
C292	0.64152	0.91494	0.52444
C293	0.66321	0.91171	0.68301
C294	0.6626	0.92943	0.86144
C295	0.64048	0.95024	0.88109
C296	0.6889	1.04807	0.62281
C297	0.73184	1.06944	0.64235
C298	0.75002	1.08691	0.82139
C299	0.72496	1.08279	0.98082
C300	0.68204	1.0616	0.96068

6. N_2 Physisorption

The nitrogen sorption isotherm of Aza-Ph-COF shows a distinct two-step profile characteristic of a highly porous material. The isotherms of Aza-Naph- and Aza-2Ph-COF exhibit an almost smooth transition between the two steps, resulting in a gradual indistinct nitrogen uptake (**Figure S25**). This is consistent with micropores, in agreement with the reduced pore size obtained from modeling. This observation is also in line with literature reports on frameworks with similar pore sizes and pore-modified COFs.[4]

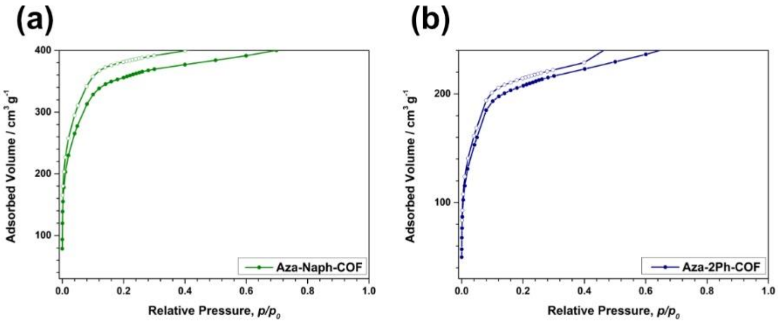

Figure S25. (**a**), (**b**) Nitrogen sorption isotherms of Aza-Naph- and Aza-2Ph-COF zoomed in in the low partial pressure range, respectively.

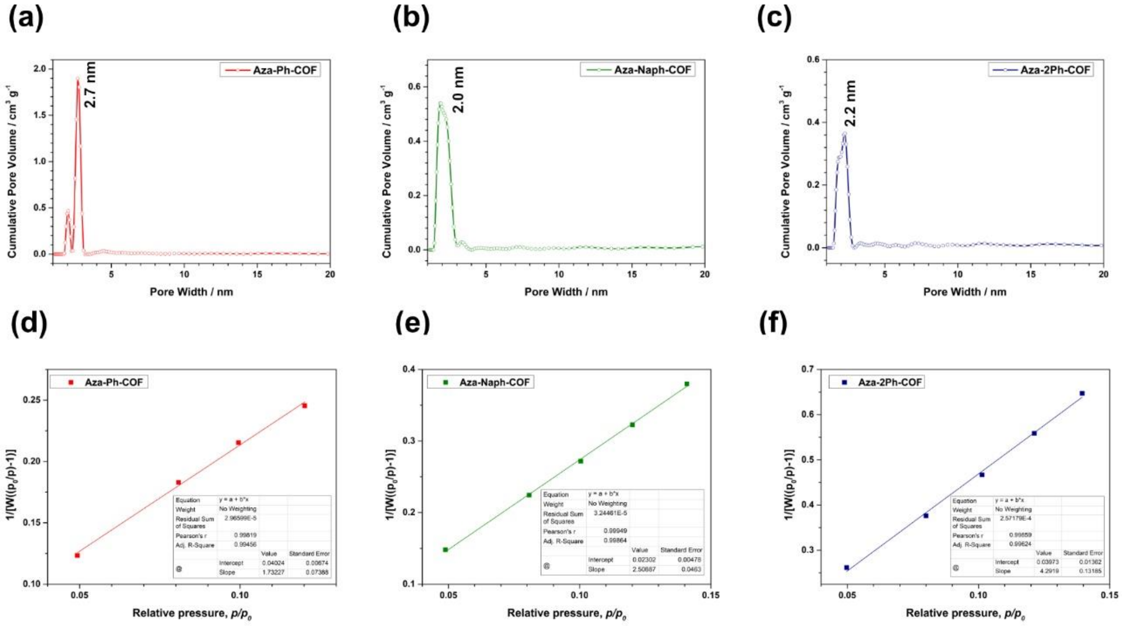

Figure S26. (**a, d**), (**b, e**), and (**c, f**) Pore size distributions and BET plots of Aza-Ph-, Aza-Naph-, and Aza-2Ph-COF, respectively.

7. Transmission electron microscopy (TEM)

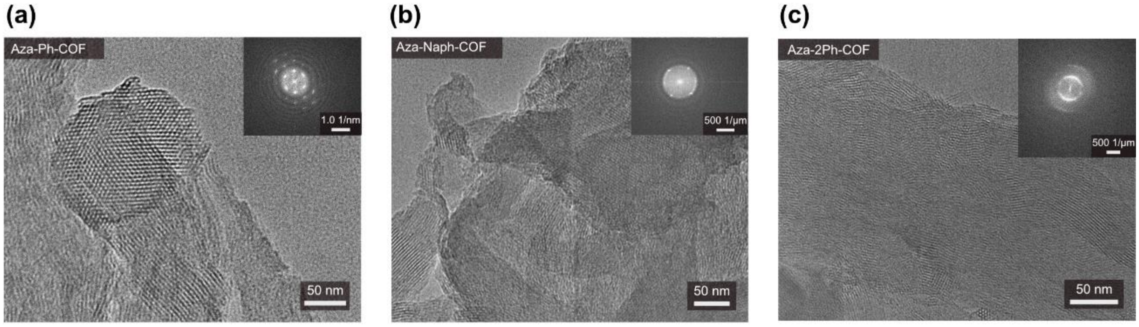

Figure S27. (**a**), (**b**), and (**c**) Transmission electron microscopy images of Aza-Ph-COF, Aza-Naph-COF, and Aza-2Ph-COF and the respective power spectra as insets, respectively.

8. Thermogravimetric Analysis (TGA)

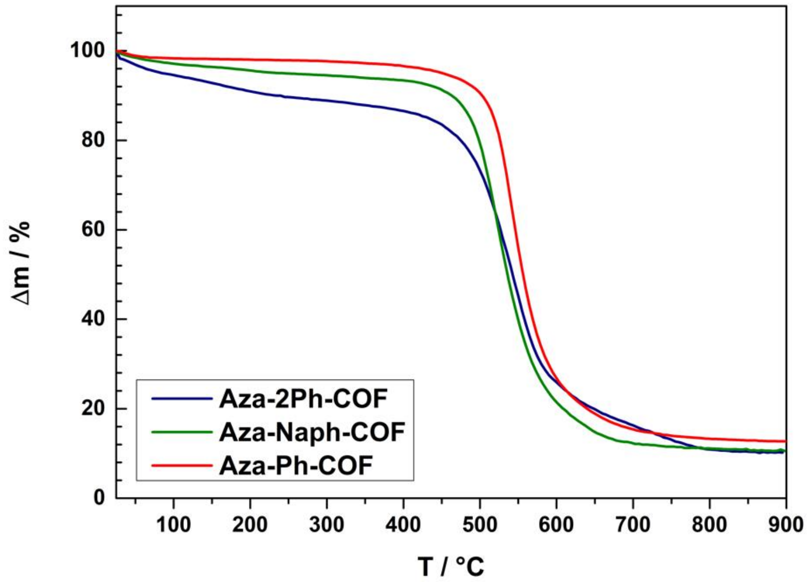

Figure S28. Thermogravimetric analysis of Aza-Ph- (red), Aza-Naph- (green), and Aza-2Ph-COF (blue) measured for bulk materials.

9. Thin film analysis

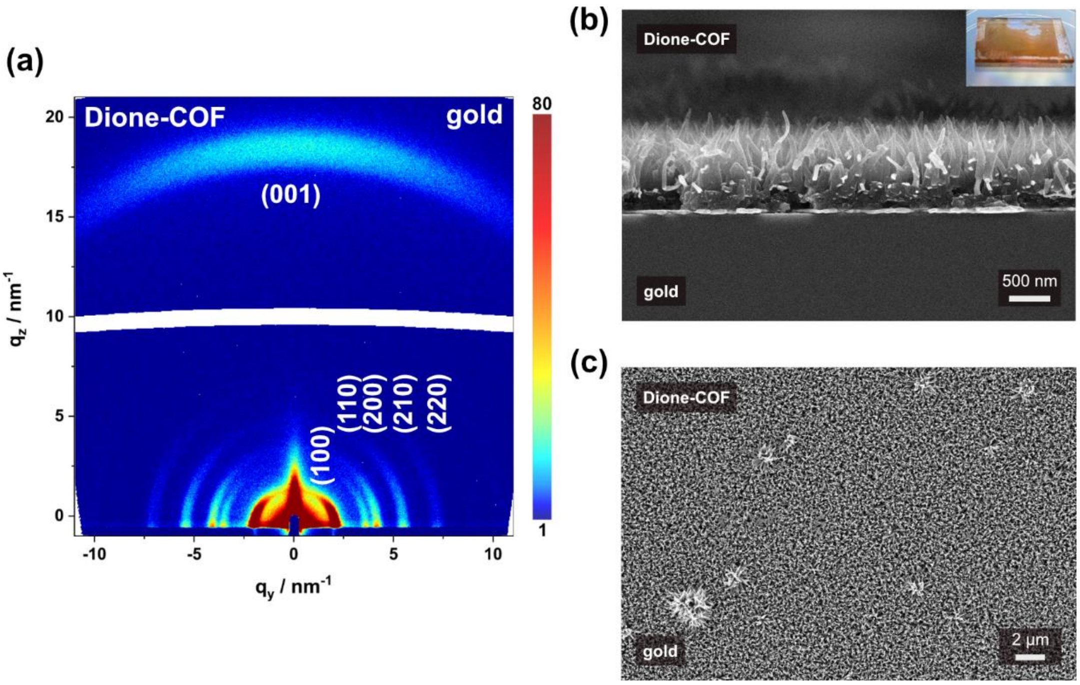

Figure S29. (**a**) GIWAXS 2D patterns of Dione-COF thin film grown on gold-coated glass substrates. (**b**), (**c**) SEM cross-section and top-view images of Dione-COF thin film grown on a gold-coated glass substrate. The inset shows a macroscopic photograph of Dione-COF thin film grown on a glass substrate.

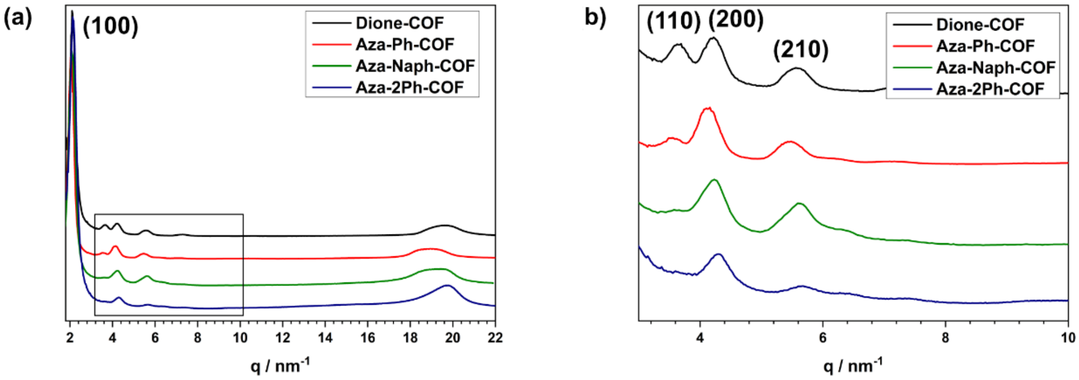

Figure S30. (**a**) Data reduction plots of the whole measured range and (**b**) a selected range between q = 5 and 10 nm^{-1} of the obtained GIWAXS data for Dione-COF (black), Aza-Ph-COF (red), Aza-Naph-COF (green), and Aza-2Ph-COF (blue). Intensity ratios of the (110) and (200) planes change for the Aza-COFs in comparison with the Dione-COF.

10. Photophysical properties

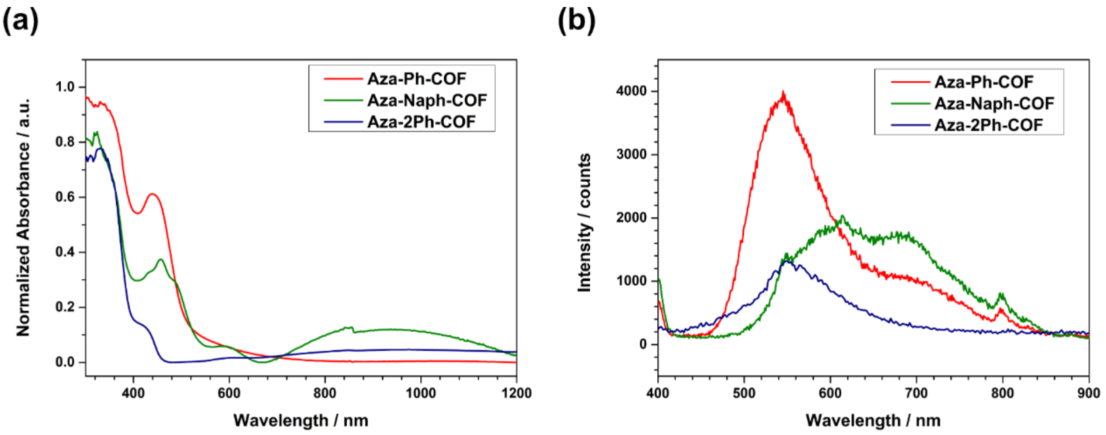

Figure S31. (**a**) Normalized absorption spectra of Aza-Ph-COF (red), Aza-Naph- (green) and Aza-2Ph-COF (blue) thin films on quartz substrates. (**b**) Normalized PL emission spectra of Aza-Ph- (red), Aza-Naph-COF (green) and Aza-2Ph-COF (blue) thin films on quartz substrates.

11. Transient absorption measurements

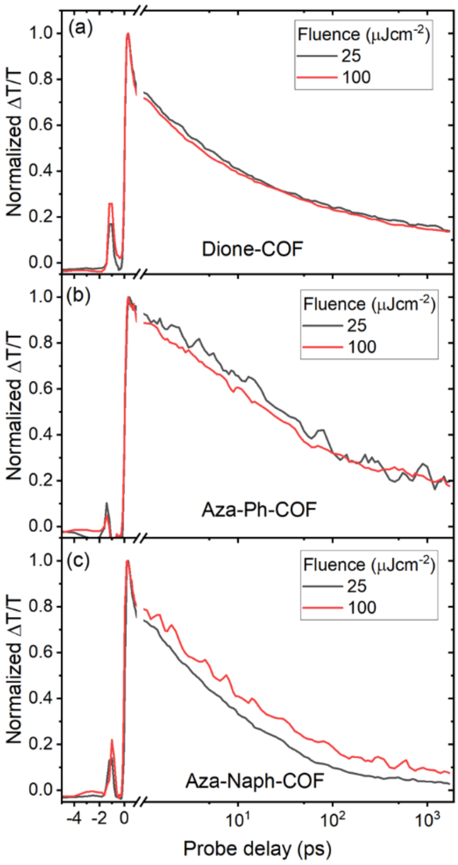

Figure S32. (**a**), (**b**), and (**c**) TA kinetics monitored at 750–770 nm for Dione-COF, Aza-Ph-COF and Aza-Naph-COF for high and low excitation fluences, respectively. Comparing high and low fluence kinetics, no significant changes in the TA dynamics can be observed allowing us to rule out exciton-exciton annihilation as a major decay pathway of the excitons.

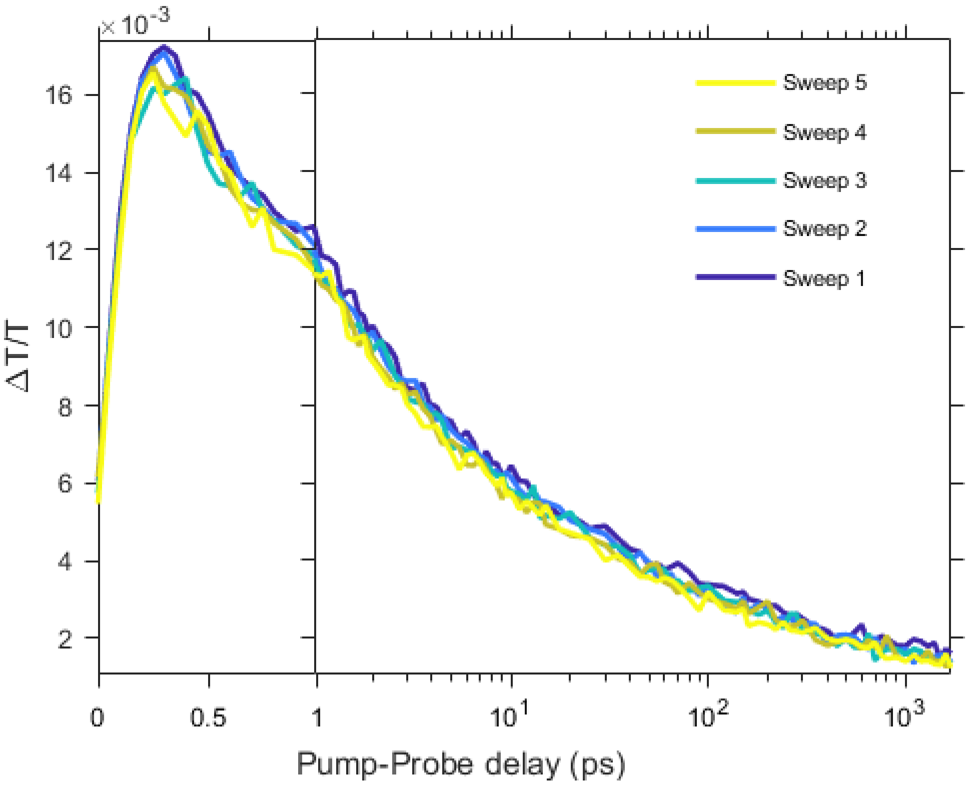

Figure S33. TA kinetics at 750–770 nm for Dione-COF film for 5 consecutive (1 sweep represents 1 experiment) TA experiments (excitation fluence: 100 µJ/cm^2) on the same spot. The signal intensity does not change for consecutive TA experiments, meaning the films do not degrade during measurements. Similar conclusions can be drawn for Aza-Ph- and Aza-Naph-COF films.

12. References

[1] J. C. Walsh, K.-L. M. Williams, D. Lungerich, G. J. Bodwell, *Eur. J. Org. Chem.* **2016**, *2016*, 5933.

[2] J. Merz, M. Dietz, Y. Vonhausen, F. Wöber, A. Friedrich, D. Sieh, I. Krummenacher, H. Braunschweig, M. Moos, M. Holzapfel, C. Lambert, T. B. Marder, *Chem. Eur. J.* **2020**, *26*, 438.

[3] L. M. Salonen, D. D. Medina, E. Carbó-Argibay, M. G. Goesten, L. Mafra, N. Guldris, J. M. Rotter, D. G. Stroppa, C. Rodríguez-Abreu, *Chem. Commun.* **2016**, *52*, 7986.

[4] a) A. Nagai, Z. Guo, X. Feng, S. Jin, X. Chen, X. Ding, D. Jiang, *Nat. Commun.* **2011**, *2*, 536; b) W. Meng, Y. Zeng, Z. Liang, W. Guo, C. Zhi, Y. Wu, R. Zhong, C. Qu, R. Zou, *ChemSusChem* **2018**, *11*, 3751.
